# Nkx6.1 Controls a Gene Regulatory Network Required for Establishing and Maintaining Pancreatic Beta Cell Identity

**DOI:** 10.1371/journal.pgen.1003274

**Published:** 2013-01-31

**Authors:** Ashleigh E. Schaffer, Brandon L. Taylor, Jacqueline R. Benthuysen, Jingxuan Liu, Fabrizio Thorel, Weiping Yuan, Yang Jiao, Klaus H. Kaestner, Pedro L. Herrera, Mark A. Magnuson, Catherine Lee May, Maike Sander

**Affiliations:** 1 Department of Pediatrics and Cellular and Molecular Medicine, University of California San Diego, La Jolla, California, United States of America; 2 Department of Pathology and Laboratory Medicine, Children's Hospital of Philadelphia, Philadelphia, Pennsylvania, United States of America; 3 Department of Genetic Medicine and Development, University of Geneva Faculty of Medicine, Geneva, Switzerland; 4 Center for Stem Cell Biology, Vanderbilt University, Nashville, Tennessee, United States of America; 5 Department of Genetics and Institute for Diabetes, Obesity and Metabolism, University of Pennsylvania, Philadelphia, Pennsylvania, United States of America; University of Copenhagen, Denmark

## Abstract

All pancreatic endocrine cell types arise from a common endocrine precursor cell population, yet the molecular mechanisms that establish and maintain the unique gene expression programs of each endocrine cell lineage have remained largely elusive. Such knowledge would improve our ability to correctly program or reprogram cells to adopt specific endocrine fates. Here, we show that the transcription factor Nkx6.1 is both necessary and sufficient to specify insulin-producing beta cells. Heritable expression of *Nkx6.1* in endocrine precursors of mice is sufficient to respecify non-beta endocrine precursors towards the beta cell lineage, while endocrine precursor- or beta cell-specific inactivation of *Nkx6.1* converts beta cells to alternative endocrine lineages. Remaining insulin^+^ cells in conditional *Nkx6.1* mutants fail to express the beta cell transcription factors Pdx1 and MafA and ectopically express genes found in non-beta endocrine cells. By showing that Nkx6.1 binds to and represses the alpha cell determinant *Arx*, we identify *Arx* as a direct target of Nkx6.1. Moreover, we demonstrate that Nkx6.1 and the *Arx* activator Isl1 regulate *Arx* transcription antagonistically, thus establishing competition between Isl1 and Nkx6.1 as a critical mechanism for determining alpha versus beta cell identity. Our findings establish Nkx6.1 as a beta cell programming factor and demonstrate that repression of alternative lineage programs is a fundamental principle by which beta cells are specified and maintained. Given the lack of Nkx6.1 expression and aberrant activation of non-beta endocrine hormones in human embryonic stem cell (hESC)–derived insulin^+^ cells, our study has significant implications for developing cell replacement therapies.

## Author Summary

Diabetes is a disease caused by the loss or dysfunction of insulin-producing beta cells in the pancreas. Recent studies suggest that modification of the beta cells' differentiation state is among the earliest events marking the progressive failure of beta cells in diabetes. Currently, very little is known about the factors that instruct cells to adopt beta cell characteristics and maintain the differentiated state of beta cells. We have discovered that a single transcription factor can instruct precursor cells of other endocrine cell types to change their identity and differentiate into beta cells. Conversely, inactivation of the transcription factor in endocrine precursors prevents their differentiation into beta cells and results in excess production of other endocrine cell types. When the factor is specifically inactivated in beta cells, beta cells lose their identity and adopt characteristics of other endocrine cell types, similar to what is seen in animal models of diabetes. Thus, we have identified a single factor that is both sufficient to program beta cells and necessary for maintaining their differentiated state. This factor could be an important target for diabetes therapy and could help reprogram other cell types into beta cells.

## Introduction

Innovative strategies for diabetes therapy aim to replace lost insulin-producing beta cells by reprogramming other cell types or by deriving beta cells from pluripotent cells. Ectopic expression of the transcription factors Pdx1, Neurogenin 3 (encoded by the *Neurog3* gene; Ngn3), and MafA has been shown to reprogram pancreatic exocrine acinar cells into beta-like cells [1]. Similarly, some success in reprogramming of liver cells into beta cells has been reported after misexpression of Pdx1, Ngn3, MafA, NeuroD, or Nkx6.1 [2]–[6]. Moreover, recent studies have demonstrated that pancreatic endocrine alpha cells can spontaneously convert into beta cells after near complete ablation of beta cells in adult mice [7]. Conversely, loss of beta cell identity and partial conversion of beta cells into other endocrine cell types has recently been identified as an early event marking beta cell failure in diabetes [8]. Thus, substantial plasticity exists between pancreatic cell types, and this plasticity could potentially be exploited to halt diabetes progression or to replenish beta cells in diabetic individuals. However, little is still known about the factors that control this plasticity.

During embryonic development, all endocrine cell types are derived from a common endocrine precursor population marked by the transcription factor Ngn3 [9], [10]. Ngn3 activity is required for the specification of all endocrine cells [11] and the expression of Arx and Pax4, two transcription factors that control endocrine subtype choices downstream of *Ngn3*. *Arx*-deficient mice display a loss of alpha cells and concomitant increase in beta and delta cells, while *Pax4-*deficiency results in the opposite phenotype of reduced beta and delta cells but increased alpha cells [12], [13]. Strikingly, forced expression of *Pax4* in endocrine precursors and their differentiated progeny imparts a beta-like cell identity to differentiating precursors, resulting in hyperplastic islets with an excess of beta-like cells at the expense of the other endocrine cell types [14]. However, despite their increased beta cell mass, mice misexpressing *Pax4* eventually become diabetic and succumb prematurely, suggesting that sustained expression of Pax4 is not compatible with normal beta cell function. Since Pax4 is normally absent from beta cells and only transiently expressed in endocrine precursors during embryogenesis [15], it is possible that proper beta cell development and maturation requires Pax4 downregulation. Similar to Pax4, misexpression of Pdx1 in endocrine precursors has also been shown to favor a beta cell fate choice over other endocrine cell types [16]. Unlike ectopic Pax4 expression, forced expression of Pdx1 did not reduce the numbers of delta and PP cells, but selectively affected the ratio between beta and alpha cells. Therefore, Pdx1 activity appears to primarily control the alpha *versus* beta cell fate decision, which is consistent with its expression in both beta and delta cells [13]. Nkx2.2 has recently been identified as a beta cell maintenance factor and stabilizes beta cell fate by repressing the alpha cell fate determinant *Arx* [17]. While these studies have provided insight into the factors involved in endocrine cell type specification and maintenance, still little is known about how these factors interact to establish and maintain gene expression programs characteristic of each endocrine cell type. In particular, it is unclear which molecular mechanisms operate in beta cell precursors to ensure that alternative endocrine lineage programs are repressed, while beta cell-specific programs are activated. Given the simultaneous initiation of multiple endocrine subtype programs in one cell with current human embryonic stem cell (hESC) differentiation protocols [18], [19], such knowledge is critical for refining these protocols to support the differentiation of mature and functional beta cells *in vitro*.

In addition to MafA and Mnx1 (also called Hb9) [20]–[22], in the adult pancreas Nkx6.1 is among the few transcription factors exclusively detected in beta cells. During development, Nkx6.1 is first expressed in multipotent pancreatic progenitors, where it specifies an endocrine identity by repressing the pre-acinar transcription factor Ptf1a [23]. At later developmental stages, Nkx6.1 expression persists in common progenitor cells for the ductal and endocrine cell lineages before becoming eventually restricted to the beta cell lineage [24]. Whether or not Nkx6.1 plays a role in beta cell specification and maintenance remains unknown, largely due to the lack of appropriate genetic models to study this question. Excessive early acinar cell specification and reduced numbers of Ngn3^+^ cells in *Nkx6.1* null mutant mice preclude their utility for such studies.

To determine the function of Nkx6.1 in endocrine cell type specification and beta cell maintenance, we generated novel genetic mouse models to conditionally inactivate or misexpress *Nkx6.1* after endocrine precursors have been specified. Our studies reveal that Nkx6.1 is both necessary and sufficient to specify the beta cell lineage. Nkx6.1 prevents alpha cell specification in cooperation with Pdx1 by directly repressing *Arx* through competition with the *Arx* gene activator Isl1. Furthermore, inactivation of *Nkx6.1* in beta cells causes loss of beta cell identity and conversion into delta cells. Our findings identify Nkx6.1 as a beta cell programming factor and uncover a transcriptional network that initiates and maintains beta cell-specific gene expression programs, while repressing programs of alternative endocrine lineages.

## Results

### Heritable expression of *Nkx6.1* in endocrine precursors favors a beta cell fate choice at the expense of other islet cell types

**Figure 1:**
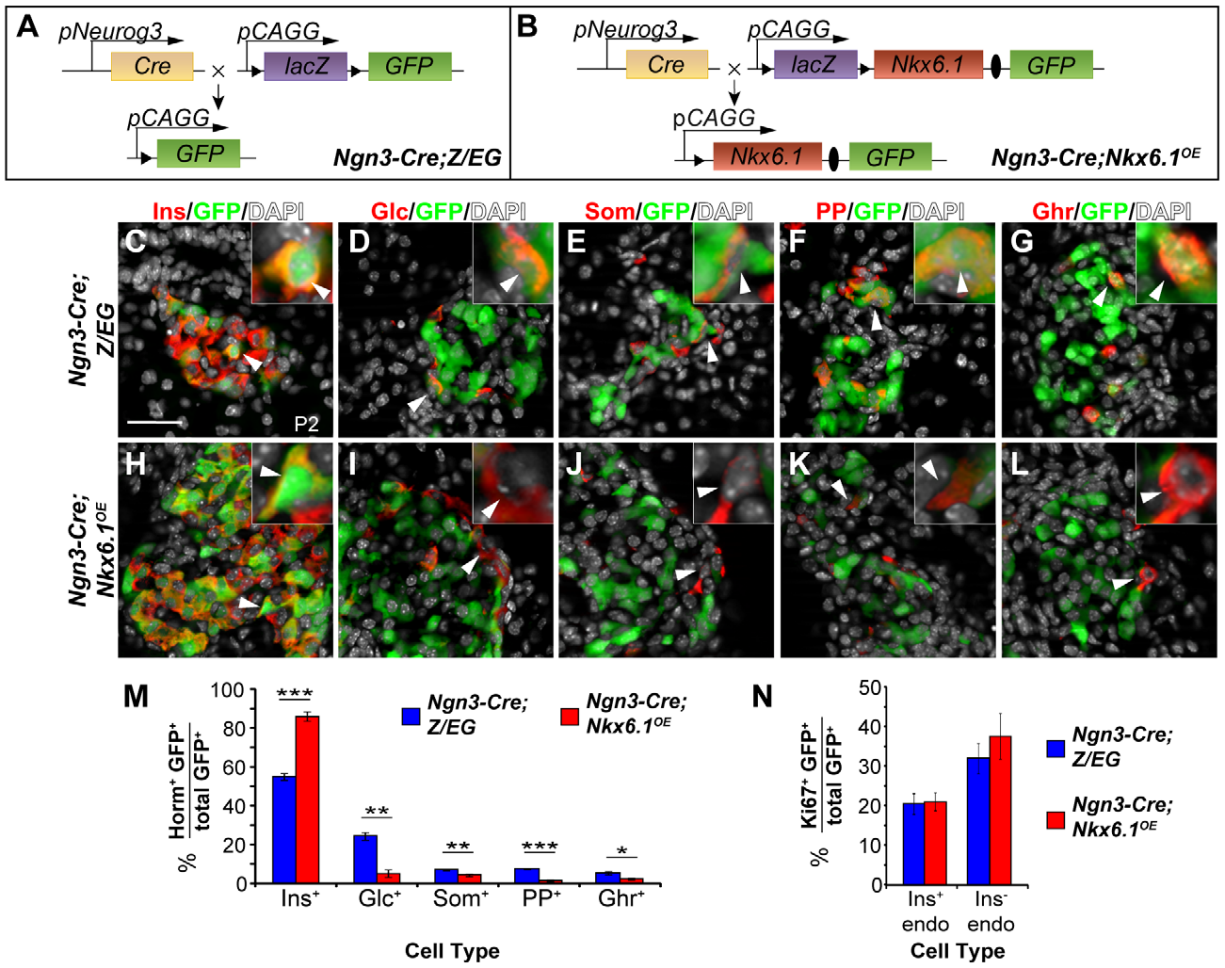
Forced *Nkx6.1* expression favors the beta cell fate choice. (A, B) Schematic of the transgenes for conditional *Nkx6.1* misexpression and cell lineage tracing; Triangles, *loxP* sites; Ovals, *internal ribosomal entry site* (IRES). (C–L) Immunofluorescence staining of pancreata from *Ngn3-Cre;Z/EG* and *Ngn3-Cre;Nkx6.1^OE^* mice at postnatal day (P) 2 for GFP together with each of the five endocrine hormones. The insets display higher magnification images. Arrowheads point to GFP^+^ cells expressing each of the five hormones in *Ngn3-Cre;Z/EG* mice and insulin, but not glucagon, somatostatin, pancreatic polypeptide, or ghrelin in *Ngn3-Cre;Nkx6.1^OE^* mice. Quantification of hormone^+^GFP^+^ (M) or Ki67^+^GFP^+^ (N) co-positive cells as a percentage of all GFP-expressing cells in pancreata of *Nkx6.1^f/−^;Ngn3-Cre;Z/EG* and *Ngn3-Cre;Z/EG* mice at P2 (n = 4). Forced expression of *Nkx6.1* in endocrine precursors favors a beta cell fate choice over all other non-beta endocrine cell fate choices. Horm, hormones; Ins, insulin; Glc, glucagon; Som, somatostatin; PP, pancreatic polypeptide; Ghr, ghrelin; endo, endocrine. Scale bar = 50 µm. Error bars represent S.E.M; *p<0.05, **p<0.01, ***p<0.001.

During pancreatic development, Nkx6.1 is expressed in a subset of Ngn3^+^ cells and is then exclusively maintained in beta cells [25], suggesting that the specification of non-beta endocrine cell types might require *Nkx6.1* downregulation. To explore whether expression of *Nkx6.1* in all, or at least the majority of, Ngn3^+^ cells is sufficient to allocate precursors to the beta cell lineage, we heritably expressed Nkx6.1 in Ngn3^+^ cells, utilizing a mouse line that allows for conditional overexpression of Nkx6.1 after expression of Cre recombinase (*Nkx6.1^OE^* mice). In *Nkx6.1^OE^* mice, concomitant expression of Nkx6.1 and enhanced green fluorescent protein (eGFP) is induced by Cre recombinase-mediated excision of a *lacZ* expression cassette flanked by *loxP* sites. The *Nkx6.1^OE^* transgene design is analogous to the *Z/EG* transgene, in which Cre recombinase induces expression of GFP by recombining *loxP* sites flanking a *lacZ* cassette. Therefore, *Z/EG* mice were used as controls for the *Nkx6.1^OE^* strain (Figure 1A, 1B). We induced transgene recombination with *Ngn3-Cre* and compared the relative contribution of recombined GFP^+^ cells to each of the five endocrine cell types in *Ngn3-Cre;Nkx6.1^OE^* and *Ngn3-Cre;Z/EG* control mice. Notably, due to mosaic expression of the transgenes, not all hormone^+^ cells expressed GFP. At postnatal day (P) 2, 55.1±1.7% of the GFP^+^ cells expressed insulin in *Ngn3-Cre;Z/EG* control mice, while 86.1±2.5% of the GFP^+^ cells were insulin^+^ in *Ngn3-Cre;Nkx6.1^OE^* mice (Figure 1C, 1H, 1M; *P*<0.001), suggesting that Nkx6.1 favors a beta cell fate choice. Consistent with this notion, *Nkx6.1*-expressing endocrine precursor cells displayed a significantly decreased propensity to differentiate into glucagon^+^, somatostatin^+^, pancreatic polypeptide (PP)^+^, or ghrelin^+^ cells compared to endocrine precursor cells from control mice (Figure 1D–1G, 1I–1M). Since *Nkx6.1* expression did not affect cell replication, as shown by analysis of the proliferation marker Ki67 (Figure 1N), or survival [23], these data indicate that Nkx6.1 promotes beta cell differentiation from endocrine precursors at the expense of alternative endocrine fates.

Similar to Nkx6.1, conditional expression of *Pax4* in mouse endocrine precursors results in beta cell specification at the expense of all other endocrine cell types [14]. The expression of Pax4 leads to oversized islets and is eventually accompanied by beta cell dysfunction and diabetes. To determine whether transgenic *Nkx6.1* expression similarly causes islet and beta cell hyperplasia, we compared islet size in *Ngn3-Cre;Nkx6.1^OE^* and control mice at P2 and at 5 months of age. Consistent with our observation that Nkx6.1 overexpression in adult beta cells does not stimulate beta cell expansion [26], *Ngn3-Cre;Nkx6.1^OE^* mice displayed normal islet cell mass (Figure S1A, S1B). Furthermore, 5-month-old *Ngn3-Cre;Nkx6.1^OE^* mice exhibited normal glucose tolerance (Figure S1C), showing that sustained expression of the *Nkx6.1* transgene does not perturb glucose homeostasis.

To next explore the extent of endocrine precursor reprograming and to assess the maturity of beta cells in *Ngn3-Cre;Nkx6.1^OE^* mice, we analyzed insulin^+^ progeny of targeted endocrine precursors for the expression of critical beta cell markers and possible ectopic expression of non-beta endocrine cell markers. As expected, insulin^+^GFP^+^ cells in *Ngn3-Cre;Nkx6.1^OE^* mice expressed the beta cell marker Pdx1, MafA, and Pax6 at P2, showing that transgenic *Nkx6.1* expression in endocrine precursors and their progeny does not impair beta cell maturation (Figure S1D–S1I). Next, to determine whether Nkx6.1 is sufficient to fully repress alternative endocrine lineage programs during endocrine cell differentiation, we analyzed insulin^+^ cells in *Ngn3-Cre;Nkx6.1^OE^* mice for expression of Arx, Brn4, glucagon, and somatostatin. At P2, targeted GFP^+^ cells in *Ngn3-Cre;Nkx6.1^OE^* mice were largely indistinguishable from their counterparts in control mice and rarely displayed coexpression of insulin with any of these non-beta endocrine markers (Figure S1J–S1Q; arrowheads), suggesting that Nkx6.1 is effective in fully establishing a beta cell expression program.

### Forced *Nkx6.1* expression is not sufficient to reprogram differentiated non-beta endocrine cells into beta cells

We next sought to determine whether *Nkx6.1* acts by inducing cell fate conversion during differentiation of Ngn3^+^ endocrine precursors or by converting already differentiated non-beta endocrine cells. Since we observed residual glucagon^+^, somatostatin^+^, pancreatic polypeptide (PP)^+^, and ghrelin^+^ cells misexpressing Nkx6.1 at P2 (Figure 1M), we first examined whether these cells convert into beta cells during postnatal life, as observed after Pdx1 expression in Ngn3^+^ cells [16]. Different from *Ngn3-Cre;Pdx1^OE^* mice, we found that targeted non-beta endocrine cells persisted in 5-month-old *Ngn3-Cre;Nkx6.1^OE^* mice (Figure S2A–S2H). Notably, the endocrine cell type ratios observed in *Ngn3-Cre;Nkx6.1^OE^* mice at P2 are largely maintained at 5 months of age (Figure S2I), suggesting that additional non-beta-to-beta cell fate conversion does not occur postnatally. To directly test whether forced expression of Nkx6.1 in differentiated alpha cells triggers their conversion into beta cells, we induced recombination of the *Nkx6.1^OE^* transgene with *Glucagon-Cre (Glc-Cre)*. Consistent with the persistence of targeted non-beta endocrine cells in adult *Ngn3-Cre;Nkx6.1^OE^* mice, we failed to observe insulin^+^ cells expressing GFP (Figure S3). These findings pinpoint Nkx6.1 beta cell programming activity to a period between the Ngn3^+^ state and activation of hormone gene expression.

### *Nkx6.1* is required for beta cell specification downstream of *Ngn3*

Since global loss of *Nkx6.1* impairs the generation of Ngn3^+^ endocrine precursors [23], it has remained unclear whether beta cell development requires Nkx6.1 activity downstream of *Ngn3*. To investigate a potential requirement for *Nkx6.1* in this process, we constructed a conditional mutant allele for *Nkx6.1* by flanking exon 2 with *loxP* sites (Figure S4A). Cre recombinase-mediated deletion of exon 2 eliminates a large portion of the DNA-binding homeodomain and additionally introduces a frameshift, resulting in three premature stop codons in exon 3, which cause termination of translation [27]. Importantly, mice heterozygous or homozygous for the *Nkx6.1^flox^* (*Nkx6.1^f^*) allele show no abnormalities, suggesting that the floxed allele of *Nkx6.1* is fully functional. To verify that Cre-mediated recombination of the *Nkx6.1^f^* allele generates a null allele, we intercrossed *Nkx6.1^f/+^;Prm1-Cre* and *Nkx6.1^+/−^* mice to induce recombination of the *Nkx6.1^f^* allele in germ cells (*Nkx6.1^Δf/−^* allele). As expected, these *Nkx6.1^Δf/−^* mice phenocopied *Nkx6.1* germline null mutant mice [28], and died immediately after birth, manifesting paralysis of their upper extremities and asphyxia (Figure S4B). Western blot analysis of Nkx6.1 protein expression in pancreata from *Nkx6.1^Δf/−^* embryos at embryonic day (e) 14.5 showed a complete absence of Nkx6.1 (Figure S4C). The pancreas of *Nkx6.1^Δf/−^* embryos was of normal size, but displayed a drastic reduction in insulin^+^ cells at e18.5 (Figure S4D–S4F), phenocopying *Nkx6.1* ^−/−^ mice [29].

**Figure 2:**
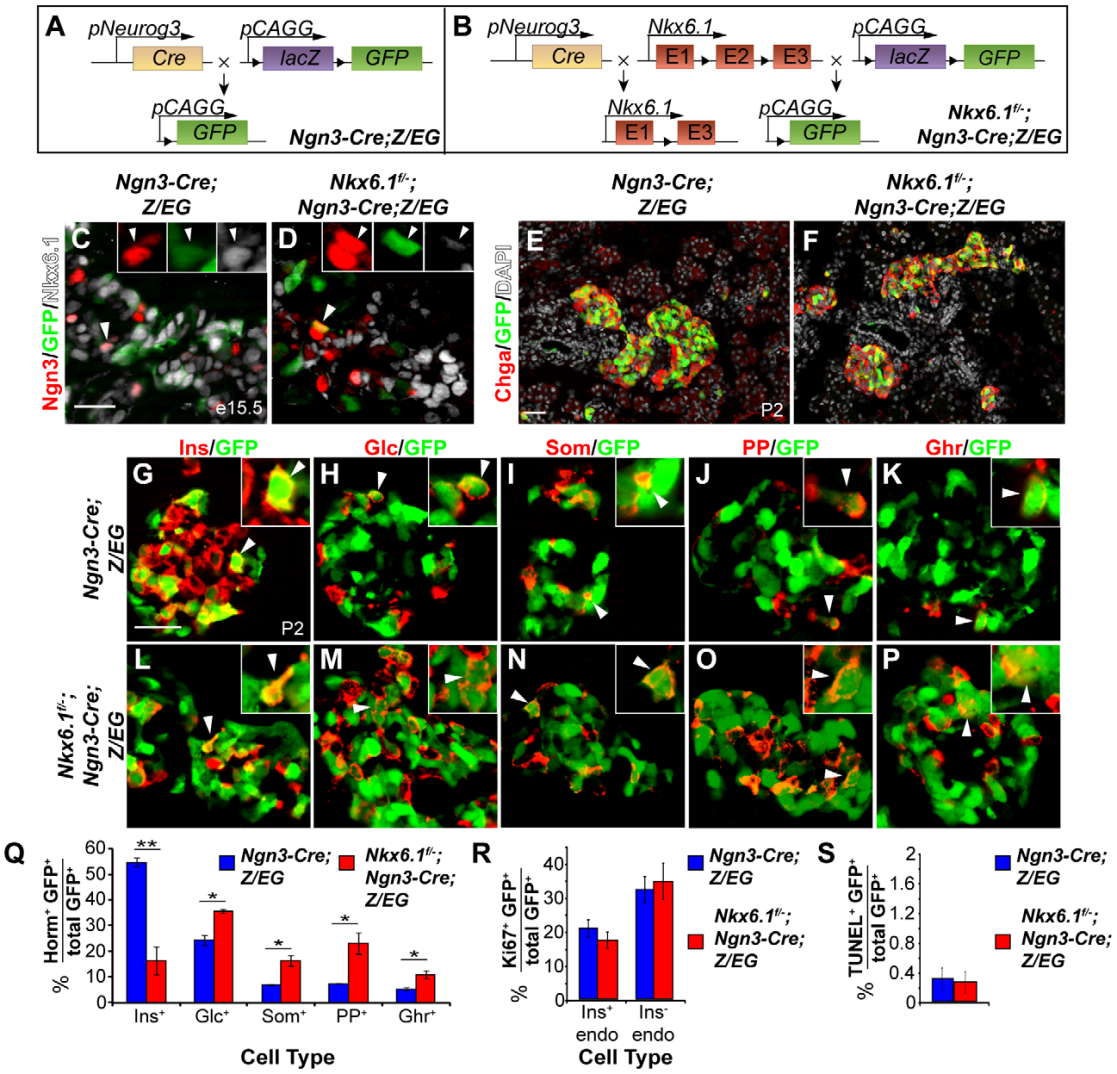
*Nkx6.1* is required for beta cell specification downstream of Ngn3. (A, B) Schematic of the alleles and transgenes for *Nkx6.1* inactivation and lineage tracing; Triangles, *loxP* sites. Immunofluorescence staining of pancreata at e15.5 (C, D) or postnatal day (P) 2 (E–P). Recombined, GFP^+^ cells are restricted to the endocrine compartment (antibody against the pan-endocrine marker Chromogranin A, Chga) in control (E) and *Nkx6.1^f/−^;Ngn3-Cre;Z/EG* mice (F). The insets show higher magnifications and arrowheads point to GFP^+^ cells expressing Ngn3 (C, D) or hormones (G–P). Quantification of hormone^+^GFP^+^ (Q), Ki67^+^GFP^+^ (R), or TUNEL^+^GFP^+^ (S) co-positive cells as a percentage of all GFP-expressing cells in pancreata of *Nkx6.1^f/−^;Ngn3-Cre;Z/EG* and *Ngn3-Cre;Z/EG* mice at P2 (n = 4). Loss of *Nkx6.1* in endocrine precursors favors alternative, non-beta endocrine cell fate choices over beta cell fate. Horm, hormones; Ins, insulin; Glc, glucagon; Som, somatostatin; PP, pancreatic polypeptide; Ghr, ghrelin; endo, endocrine. Scale bar = 50 µm. Error bars represent S.E.M; *p<0.05, **p<0.01.

To determine whether *Nkx6.1* is required for beta cell formation from Ngn3^+^ precursors, we utilized *Ngn3-Cre* to simultaneously induce recombination of the *Nkx6.1^f^* allele and the *Z/EG* reporter transgene for stable lineage tracing of all progeny derived from Ngn3^+^ cells. In *Nkx6.1^f/−^;Ngn3-Cre;Z/EG* embryos, Cre recombines the *loxP* sites in both the *Nkx6.1^f^* allele and the *Z/EG* transgene to produce cells that are deficient for *Nkx6.1* and express eGFP (Figure 2A, 2B). At e15.5, when *Ngn3* expression peaks [11], Nkx6.1 was detected in a large subset of Ngn3^+^ and GFP^+^ cells derived from the *Ngn3*-expressing domain in *Ngn3-Cre;Z/EG* control embryos (Figure 2C). In *Nkx6.1^f/−^;Ngn3-Cre;Z/EG* mice, GFP^+^ cells were devoid of Nkx6.1 (Figure 2D, Figure S5B), showing the *Ngn3-Cre* transgene efficiently deletes *Nkx6.1* in Ngn3^+^ cells and their progeny. Ngn3 was similarly expressed in *Nkx6.1^f/−^;Ngn3-Cre;Z/EG* and control embryos (Figure 2C, 2D), demonstrating that loss of *Nkx6.1* in endocrine precursors does not affect Ngn3 expression.

*Nkx6.1^f/−^;Ngn3-Cre;Z/EG* mice were born at the expected Mendelian frequency, but died within the first few days after birth from dehydration and hyperglycemia; a phenotype indicative of a beta cell defect. To determine whether loss of *Nkx6.1* affects the cell fate choice of endocrine precursors, we analyzed the fate of Ngn3^+^ cells in *Ngn3-Cre;Z/EG* and *Nkx6.1^f/−^;Ngn3-Cre;Z/EG* mice. Based on our previous finding that Nkx6.1 prevents acinar cell fate specification [23], we first examined whether loss of *Nkx6.1* in Ngn3^+^ cells allocates endocrine precursors to the acinar lineage. The ability of Ngn3^+^ cells to undergo endocrine-to-acinar cell fate conversion has been previously demonstrated in conditions of reduced *Ngn3* gene dosage or impaired Notch signaling activity [30], [31]. At P2, we found GFP^+^ cells to be exclusively restricted to endocrine islets in both *Ngn3-Cre;Z/EG* and *Nkx6.1^f/−^;Ngn3-Cre;Z/EG* mice (Figure 2E, 2F), revealing that *Nkx6.1* deletion in Ngn3^+^ cells does not cause endocrine-to-acinar fate conversion. Thus, unlike multipotent pancreatic progenitors, which adopt an acinar cell identity in the absence of Nkx6.1 activity [23], Ngn3^+^ endocrine precursors are no longer competent to activate acinar gene expression programs after deletion of *Nkx6.1*.

Because *Nkx6.1*-deficient endocrine precursors differentiate into endocrine cells (Figure 2F), we next sought to determine whether loss of *Nkx6.1* affects the relative proportion of the different endocrine cell types arising from Ngn3^+^ cells. To examine the endocrine cell fate choice of Ngn3^+^ cells, we quantified how many of the recombined *Ngn3*-expressing cells were allocated to each endocrine cell lineage by co-staining for GFP as a lineage marker of Ngn3-cell progeny together with each of the five hormones individually. At P2, 55.1±1.7% of recombined cells were insulin^+^ in *Ngn3-Cre;Z/EG* control mice, while only 16.6±5.4% of recombined cells were insulin^+^ in *Nkx6.1^f/−^;Ngn3-Cre;Z/EG* mice (Figure 2G, 2L, 2Q; *P*<0.01), suggesting that *Nkx6.1*-deficient precursors have a lower propensity to differentiate into insulin^+^ cells. To test whether *Nkx6.1*-deficient Ngn3^+^ cells instead adopt non-beta endocrine cell identities, we compared the percentage of Ngn3^+^ cells that contributed to each non-beta endocrine cell lineage in *Ngn3-Cre;Z/EG* and *Nkx6.1^f/−^;Ngn3-Cre;Z/EG* mice. *Nkx6.1^f/−^;Ngn3-Cre;Z/EG* mice at P2 exhibited significantly more glucagon^+^GFP^+^ (35.9±0.8% vs. 24.6±2.0%; *P*<0.05), somatostatin^+^GFP^+^ (16.6±2.1% vs. 7.2±0.2%; *P*<0.05), PP^+^GFP^+^ (23.3±4.1% vs. 7.5±0.1%; *P*<0.025), and ghrelin^+^GFP^+^ (11.1±1.5% vs. 5.5±0.7%; *P*<0.05) cells than *Ngn3-Cre;Z/EG* control mice (Figure 2H–2K, 2M–2Q). Together, these findings suggest that endocrine precursor cells require Nkx6.1 activity to differentiate into beta cells and that Nkx6.1 prevents precursors from adopting non-beta endocrine fates. To ascertain that the differences in islet cell type composition between *Ngn3-Cre;Z/EG* and *Nkx6.1^f/−^;Ngn3-Cre;Z/EG* mice are indeed the result of preferential precursor cell fate choices and not due to different proliferation or survival rates, we analyzed GFP^+^ cells in each genotype for their rates of proliferation and apoptosis at P2. Both insulin^+^GFP^+^ and insulin^−^GFP^+^ endocrine cells displayed similar proliferation and apoptotic rates in *Ngn3-Cre;Z/EG* and *Nkx6.1^f/−^;Ngn3-Cre;Z/EG* mice (Figure 2R, 2S), demonstrating that loss of *Nkx6.1* does not affect proliferation or survival. Together, we show that Nkx6.1 controls the fate choice between beta and non-beta endocrine cell lineages in endocrine precursor cells, without favoring any one non-beta endocrine cell type in particular.

### *Nkx6.1*-deficient insulin^+^ cells are polyhormonal and ectopically express alpha cell markers

**Figure 3:**
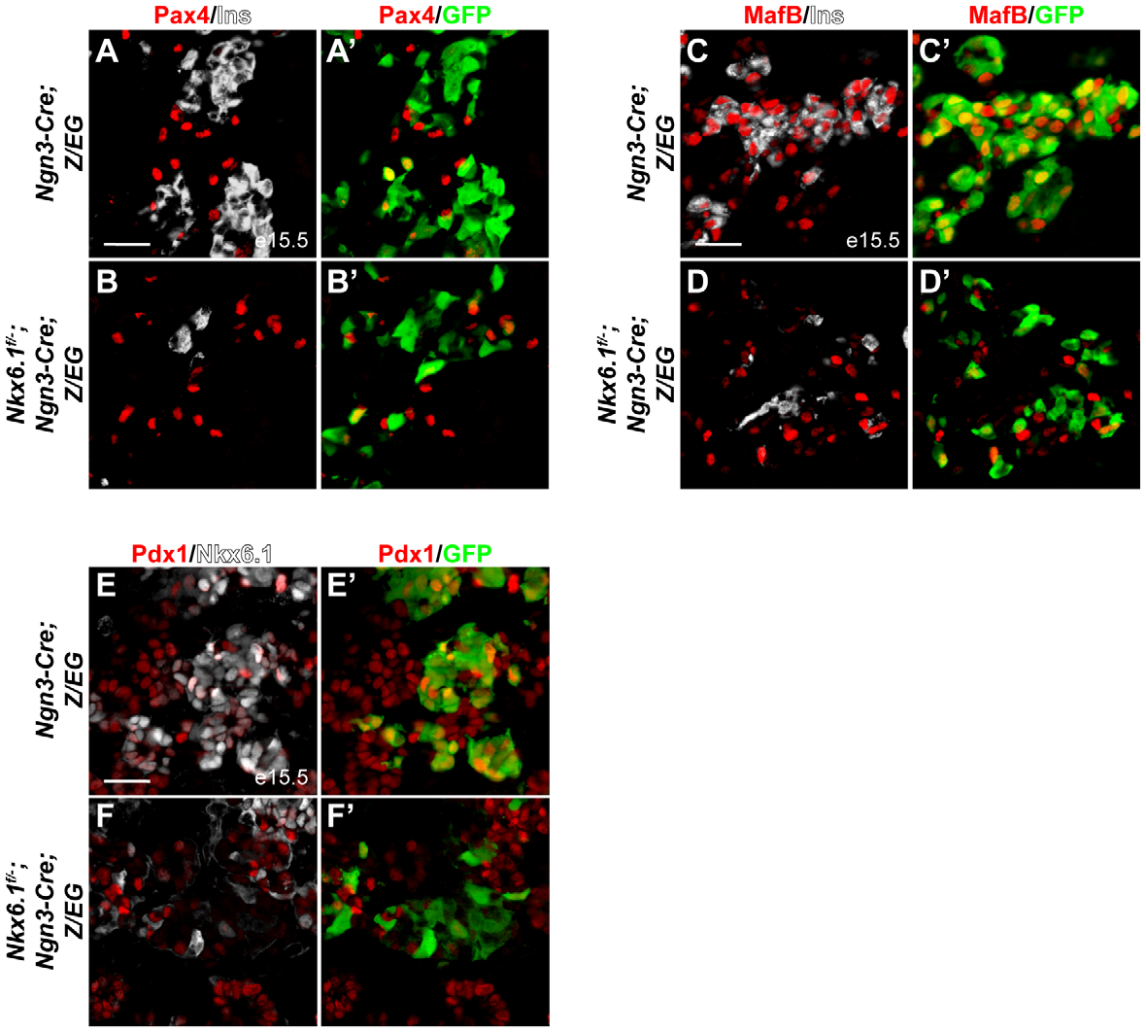
*Nkx6.1* controls Pdx1 and MafB expression. Immunofluorescence staining of pancreata at e15.5 shows no difference in Pax4 expression (A, B) in recombined, GFP^+^ cells between *Nkx6.1^f/−^;Ngn3-Cre;Z/EG* embryos and control *Ngn3-Cre;Z/EG* embryos, while MafB (C, D) and Pdx1 (E, F) expression is reduced in *Nkx6.1*-deficient, GFP^+^ cells compared to control embryos. Ins, insulin. Scale bar = 50 µm.

To investigate whether Nkx6.1 mediates beta cell specification downstream of Ngn3 by regulating transcription factors necessary for beta cell development, we analyzed expression of the beta cell progenitor markers Pax4, MafB, and Pdx1 in *Ngn3-Cre;Z/EG* and *Nkx6.1^f/−^;Ngn3-Cre;Z/EG* embryos during the peak period of beta cell differentiation at e15.5. Confirming our previous findings in *Nkx6.1* null mutant embryos [24], Pax4 expression was not affected in *Nkx6.1^f/−^;Ngn3-Cre;Z/EG* embryos (Figure 3A, 3B; 9.6% of GFP^+^ cells expressed Pax4 in control mice *vs.* 10.1% in *Nkx6.1^f/−^;Ngn3-Cre;Z/EG* mice). In contrast, the marker of newly-born alpha and beta cells, MafB, was absent from the majority of targeted cells in *Nkx6.1^f/−^;Ngn3-Cre;Z/EG* embryos (Figure 3C, 3D; 76.2% of GFP^+^ cells expressed MafB in control mice *vs.* 20.7% in *Nkx6.1^f/−^;Ngn3-Cre;Z/EG* mice), suggesting that MafB, similar to its homolog MafA [20], is controlled by Nkx6.1. Likewise, while a large percentage of Ngn3^+^ cell progeny were Pdx1^+^ in control embryos, only a small percentage expressed Pdx1 in *Nkx6.1*-deficient embryos (Figure 3E, 3F; 81.0% of GFP^+^ cells expressed Pdx1 in control mice *vs.* 18.6% of GFP^+^ cells in *Nkx6.1^f/−^;Ngn3-Cre;Z/EG* mice). Thus, Nkx6.1 controls the expression of MafB and Pdx1, but not Pax4 in embryonic beta cell precursors.

**Figure 4:**
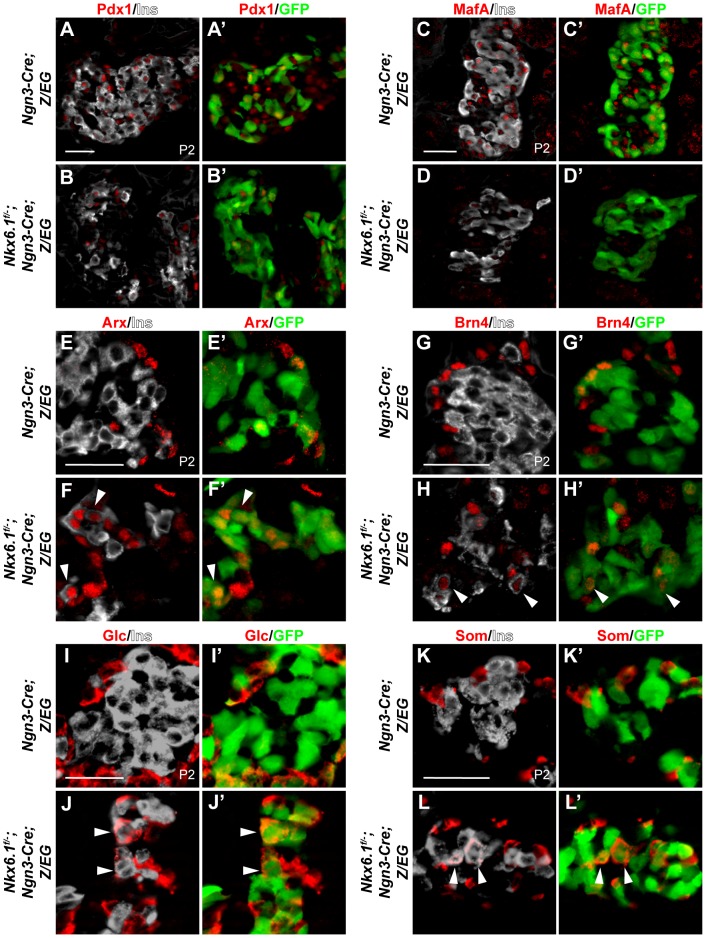
Loss of *Nkx6.1* in endocrine precursors results in activation of non-beta endocrine genes. (A–L) Immunofluorescence staining of pancreata from *Ngn3-Cre;Z/EG* and *Nkx6.1^f/−^;Ngn3-Cre;Z/EG* mice at postnatal day (P) 2 shows reduced Pdx1 (A, B), absence of MafA (C, D), and ectopic expression of Arx (E, F), Brn4 (G, H), glucagon (Glc; I, J), and somatostatin (Som; K, L) in *Nkx6.1*-deficient, recombined, insulin^+^GFP^+^ cells. Arrowheads point to insulin^+^ cells ectopically expressing non-beta endocrine markers. Ins, insulin. Scale bar = 50 µm.

Although reduced in numbers, we still observed targeted GFP^+^ cells expressing insulin in neonatal *Nkx6.1^f/−^;Ngn3-Cre;Z/EG* mice, raising the question of whether these *Nkx6.1*-deficient insulin^+^ cells properly differentiate into beta cells. To investigate how lack of Nkx6.1 affects beta cell gene expression programs, we analyzed insulin^+^ cells in neonatal *Nkx6.1^f/−^;Ngn3-Cre;Z/EG* mice for the expression of Pax6, Pdx1, and MafA. Expression of the islet cell marker Pax6 was not affected in *Nkx6.1^f/−^;Ngn3-Cre;Z/EG* mice (Figure S5C, S5D; 68.4% of insulin^+^GFP^+^ cells expressed Pax6 in control mice *vs.* 72.7% in *Nkx6.1^f/−^;Ngn3-Cre;Z/EG* mice). By contrast, confirming our findings at embryonic stages (Figure 3E, 3F), Pdx1 expression was markedly reduced in *Nkx6.1*-deficient insulin^+^ cells at P2 (Figure 4A, 4B; 76.5% of insulin^+^GFP^+^ cells expressed Pdx1 in control mice *vs.* 30.2% in *Nkx6.1^f/−^;Ngn3-Cre;Z/EG* mice). Similarly, the mature beta cell marker MafA was absent from insulin^+^ cells in *Nkx6.1^f/−^;Ngn3-Cre;Z/EG* mice (Figure 4C, 4D; 79.3% of insulin^+^GFP^+^ cells expressed MafA in control mice *vs.* 0% in *Nkx6.1^f/−^;Ngn3-Cre;Z/EG* mice). This demonstrates that beta cells require Nkx6.1 activity during their differentiation to initiate MafA expression and to maintain high levels of Pdx1. These findings are consistent with the phenotype of *Nkx6.1* null mutant mice, in which limited numbers of insulin^+^ cells lacking MafA are observed [20], [24]. We conclude that insulin expression can still be initiated in the absence of Nkx6.1, but that these insulin^+^ cells lack key features of normal beta cells.

The lack of beta cell-specific markers in *Nkx6.1*-deficient insulin^+^ cells raised the question of whether these insulin^+^ cells also carry features of alternative endocrine lineages. To determine whether loss of *Nkx6.1* in endocrine precursors results in the activation of mixed endocrine gene expression programs, we analyzed insulin^+^ cells in *Nkx6.1^f/−^;Ngn3-Cre;Z/EG* mice for coexpression of the alpha cell lineage determinants Arx and Brn4. As expected, in *Ngn3-Cre;Z/EG* control mice virtually no colocalization of Arx and Brn4 with insulin was observed at P2 (Figure 4E, 4G; 0.2% of insulin^+^GFP^+^ cells expressed Arx and 0% Brn4 in control mice). In contrast, a subset of recombined insulin^+^GFP^+^ cells in *Nkx6.1^f/−^;Ngn3-Cre;Z/EG* mice also expressed Arx and Brn4 (Figure 4F, 4H; arrowheads; 26.1% of insulin^+^GFP^+^ cells expressed Arx and 26.5% Brn4 in *Nkx6.1^f/−^;Ngn3-Cre;Z/EG* mice), showing aberrant activation of alpha cell differentiation genes. Notably, *Nkx6.1* deletion or misexpression did not affect Arx expression in the immediate progeny of endocrine precursors at e15.5 (Figure S6A–S6D), pinpointing Nkx6.1-mediated regulation of Arx to a time window between e15.5 and birth.

At P2, loss of Nkx6.1 activity was also associated with aberrant expression of glucagon in insulin^+^ cells (Figure 4I, 4J; arrowheads in J; 0.5% of insulin^+^GFP^+^ cells expressed glucagon in control mice *vs.* 29.8% in *Nkx6.1^f/−^;Ngn3-Cre;Z/EG* mice). Moreover, we found that many of the targeted insulin^+^ cells ectopically expressed somatostatin in *Nkx6.1^f/−^;Ngn3-Cre;Z/EG* mice (Figure 4K, 4L; arrowheads in L; 0% of insulin^+^GFP^+^ cells expressed somatostatin in control mice *vs.* 19.6% in *Nkx6.1^f/−^;Ngn3-Cre;Z/EG* mice). These findings demonstrate that Nkx6.1 is critical for repressing alternative endocrine lineage programs and that beta cell-specific programs can only be induced to a limited extent when *Nkx6.1* is lost.

**Figure 5:**
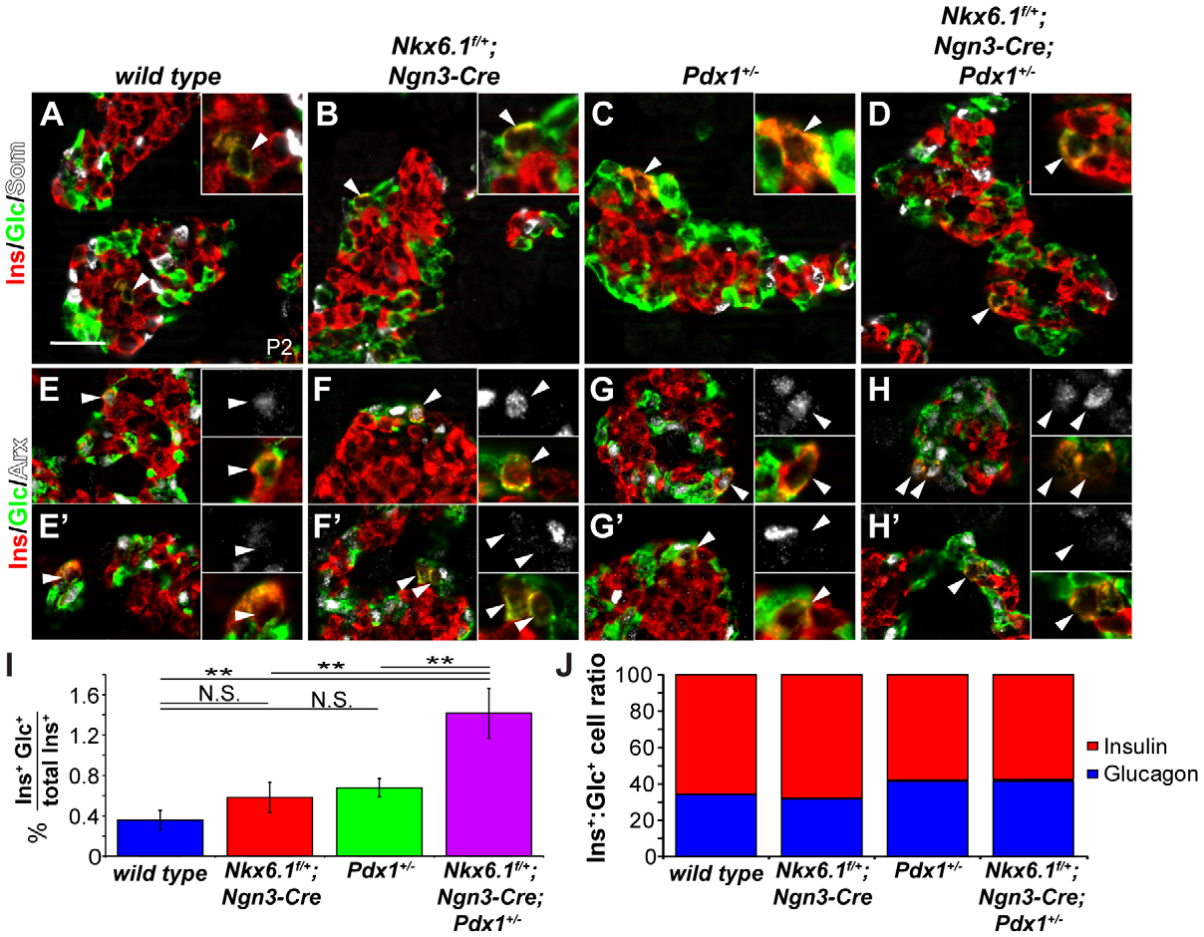
*Nkx6.1* and *Pdx1* collectively stabilize beta cell identity. (A–H) Immunofluorescence staining of pancreata from *wild type*, *Nkx6.1^f/+^;Ngn3-Cre*, *Pdx1^+/−^*, and *Nkx6.1^f/+^;Ngn3-Cre;Pdx1^+/−^* mice at postnatal day (P) 2 reveals occasional coexpression of insulin with glucagon but not with somatostatin in all genotypes (A–D; arrowheads and insets). Both Arx^+^ (E–H) and Arx^−^ (E′–H′) insulin^+^glucagon^+^ cells (arrowheads and insets) are found in all genotypes. (I) Quantification of the percentage of insulin^+^ cells co-expressing glucagon at P2 reveals significantly more insulin^+^glucagon^+^ cells in *Nkx6.1^f/+^;Ngn3-Cre*;*Pdx1* ^+/−^, *Nkx6.1^f/+^;Ngn3-Cre,* and *Pdx1^+/−^* mice compared to *wild type* controls. In addition, *Nkx6.1^f/+^;Ngn3-Cre*;*Pdx1* ^+/−^ mice show more insulin^+^glucagon^+^ cells than either single heterozygous mutant (n = 3). (J) Quantification of insulin^+^ and glucagon^+^ cell numbers in P2 pancreata shows an increase in glucagon^+^ cells in *Nkx6.1^f/+^;Ngn3-Cre;Pdx1^+/−^* and *Pdx1^+/−^* mice compared to *Nkx6.1^f/+^;Ngn3-Cre* and *wild type* mice demonstrating that loss of a single *Nkx6.1* allele does not significantly affect alpha cell numbers (n = 3). Ins, insulin; Glc, glucagon; Som, somatostatin. Scale bar = 50 µm. Error bars represent S.E.M; **p<0.01, N.S. = not significant.

Previous studies have shown that the number of glucagon^+^ cells is increased in *Pdx1* heterozygous mutant mice [32], [33]. Furthermore, beta cells lose Nkx6.1 expression upon *Pdx1* deletion in beta cells [34]. Combined with our observation that *Nkx6.1* maintains Pdx1 expression during beta cell differentiation, these findings raise the possibility that Pdx1 and Nkx6.1 cooperate through a positive feedback loop to establish and maintain beta cell identity and to repress non-beta endocrine lineage programs. To test this idea, we analyzed *wild type*, *Nkx6.1^f/+^;Ngn3-Cre, Pdx1^+/−^,* and compound heterozygous *Nkx6.1^f/+^;Ngn3-Cre;Pdx1^+/−^* mice for the ectopic expression of non-beta endocrine hormones in insulin^+^ cells at P2. While we saw no coexpression of somatostatin or PP with insulin in any of the four genotypes (Figure 5A–5D; data not shown), glucagon and insulin co-positive cells were occasionally detected in all genotypes, including *wild type* mice (Figure 5A–5D, 5I). Quantification of the percentage of insulin^+^ cells also expressing glucagon revealed significantly more dual hormone-positive cells in compound heterozygous *Ngn3-Cre;Nkx6.1^f/+^;Pdx1^+/−^* mice than in either single heterozygous mutant or in *wild type* mice (Figure 5A–5D, 5I). As previously reported [32], [33], *Pdx1* ^+/−^ mice displayed an increase in the glucagon to insulin cell ratio that was also seen in compound heterozygous *Ngn3-Cre;Nkx6.1^f/+^;Pdx1^+/−^* but not in *Nkx6.1^f/+^;Ngn3-Cre* mice (Figure 5J). Thus, haploinsufficiency for *Pdx1* but not *Nkx6.1* increases alpha cell numbers, which may reflect a non-cell autonomous effect on alpha cell proliferation, as recently shown in a mouse model of conditional deletion of *Pdx1* in beta cells [35]. To determine whether the mixed lineage identity of insulin^+^glucagon^+^ cells is associated with the expression of Arx, we co-stained pancreatic sections of mice at P2 from all genotypes for insulin, glucagon, and Arx. The majority of insulin^+^glucagon^+^ cells expressed Arx in all genotypes, although occasional insulin^+^glucagon^+^Arx^−^ cells were also detected (Figure 5E–5H; arrowheads). The observed increase in cells exhibiting mixed alpha/beta cell identity and Arx expression in *Nkx6.1*/*Pdx1* compound heterozygous mice supports the notion that Pdx1 and Nkx6.1 cooperate in beta cell fate specification by preventing activation of alpha cell-specific gene expression programs during endocrine cell differentiation.

### Nkx6.1 is a direct repressor of *Arx* and competes with the *Arx* activator Isl1 at an *Arx* intronic enhancer

**Figure 6:**
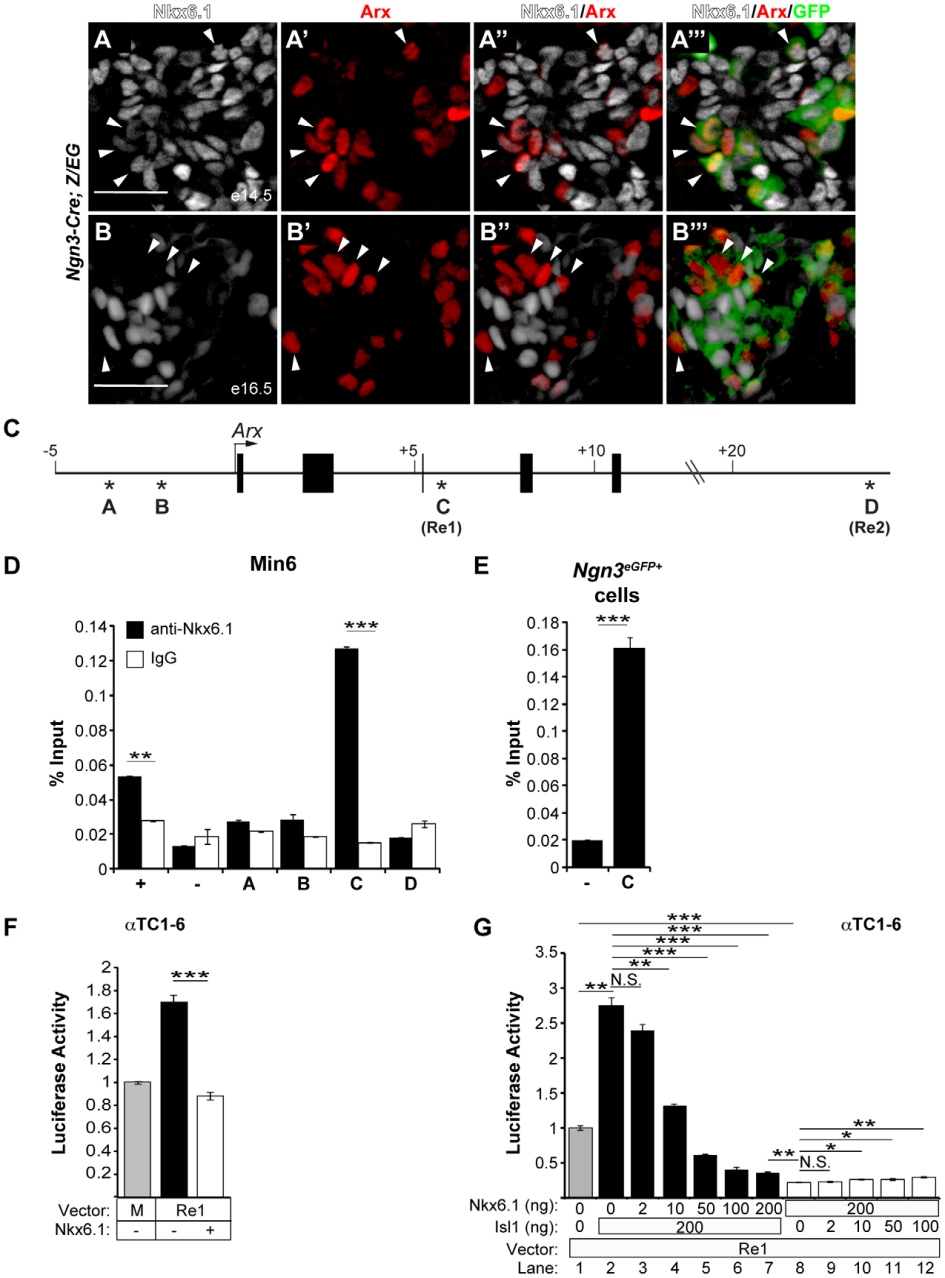
Nkx6.1 and Isl1 function as antagonistic transcriptional regulators of the *Arx Re1* enhancer. Immunofluorescence staining of pancreata from *Ngn3-Cre;Z/EG* mice at e14.5 (A) and e16.5 (B) for Nkx6.1, Arx, and GFP shows that the majority of progeny of Ngn3-expressing cells (GFP^+^) co-express Arx and Nkx6.1 at e14.5 (arrowheads in A), while the Arx^+^ and Nkx6.1^+^ domains are distinct at e16.5 (arrowheads in B point to GFP^+^Arx^+^Nkx6.1^−^ cells). (C) Schematic of the *Arx* locus; asterisks indicate phylogenetically-conserved Nkx6.1 binding motifs and numbers indicate the distance from the transcriptional start site. Nkx6.1 binds to site C (*Re1* element) in the *Arx* locus in chromatin from Min6 cells (D) and FACS-sorted GFP^+^ cells (E) from e15.5 pancreata of *Neurog3* ^eGFP^ embryos analyzed by ChIP with antibodies against Nkx6.1 or control immunoglobulin G (IgG). Mouse *glucagon* promoter and intergenic primers were used as positive (+) and negative (−) controls, respectively. (F) Co-transfection of αTC1–6 cells with the *Arx Re1* enhancer-luciferase construct, the *CMV-Renilla* expression construct, and with or without the *CMV-Nkx6.1* expression construct. Lane one (M) represents basal luciferase expression of the minimal promoter. Luciferase activity was quantified relative to the expression of the minimal promoter. Activity of the *Re1* enhancer is repressed by Nkx6.1. (G) Co-transfection of αTC1–6 cells with the *Arx Re1* enhancer-luciferase construct, *CMV-Renilla*, and with different concentration of *CMV-Nkx6.1* and *CMV-Isl1*, as indicated. Nkx6.1 prevents activation of the *Arx Re1* enhancer by Isl1 in a dose-dependent manner (lanes 2–7). Luciferase activity was quantified relative to the expression of the *Re1* enhancer. Increasing concentrations of Isl1 are not sufficient to restore baseline activity of the *Re1* enhancer in the presence of 200 ng of *CMV-Nkx6.1* (lanes 8–12). Scale bar = 50 µm. Error bars represent S.E.M; *p<0.05, **p<0.01, ***p<0.001.

To further explore how Nkx6.1 prevents endocrine precursors from adopting alpha cell identity, we examined the relationship of the Nkx6.1 and Arx expression domains in progeny of *Ngn3*-expressing cells during development. Consistent with the dependence of Arx expression on *Ngn3* [12], Arx was confined to a domain that marks descendants of *Ngn3*-expressing cells (Figure 6A′″, 6B′″; note, occasional Arx^+^GFP^−^ cells in A and B can be explained by mosaic expression of the *Z/EG* and/or *Ngn3-Cre* transgenes). At e14.5, preceding the onset of the major wave of beta cell differentiation, the majority of Arx^+^ cells also expressed Nkx6.1 and Arx^+^Nkx6.1^−^ cells were rare (Figure 6A; arrowheads). By e16.5, however, when large numbers of beta cells arise [36], GFP^+^ cells seldom coexpressed Nkx6.1 and Arx (Figure 6B; arrowheads). Thus, endocrine precursors initially activate Nkx6.1 and Arx concomitantly, but their expression domains become mutually exclusive during beta cell differentiation. Together with our finding that Nkx6.1 regulates Arx during this time window, the observed expression pattern of Arx and Nkx6.1 raised the possibility that Nkx6.1 functions as a transcriptional repressor of *Arx*.

To explore this hypothesis, we tested whether Nkx6.1 occupies *Arx* regulatory sequences and is capable of repressing *Arx* transcription. We identified two conserved Nkx6.1 binding motifs within 5 kb of the 5′ end flanking region from the *Arx* transcriptional start site (Figure 6C; site A and B) as well as 12 potential Nkx6.1 binding sites located in two previously characterized enhancers within the third intron of the *Arx* genomic sequence (Figure 6C; site C, *Re1*) and the 3′ flanking region (Figure 6C; site D, *Re2*) [37]. Recently, the *Arx Re1* and *Re2* enhancers have been shown to be required for Isl1-mediated activation of *Arx* in alpha cells, suggesting that these two enhancers are critical for *Arx* transcription [37]. Chromatin immunoprecipitation (ChIP) analyses for Nkx6.1 revealed that Nkx6.1 directly and specifically associates with the *Arx Re1* enhancer (site C) in the Min6 beta cell line (Figure 6D) and in embryonic endocrine precursors isolated by fluorescence-activated cell sorting (FACS) from *Ngn3^eGFP/+^* embryonic pancreata at e15.5 (Figure 6E). Discordant with a previous report, which reported binding of Nkx6.1 to site B in a beta cell line [17], no association was observed with the other sites (sites A, B, or D) containing Nkx6.1 motifs (Figure 6D). Transfection of the αTC1–6 alpha cell line with an expression plasmid for *Nkx6.1* and a luciferase reporter construct containing the *Arx Re1* enhancer sequence revealed that Nkx6.1 significantly reduced reporter gene activity (Figure 6F). Confirming previous findings [37], transfection of an expression plasmid for *Isl1* activated the *Re1* enhancer (Figure 6G). Co-transfection of *CMV-Nkx6.1* abolished the ability of *Isl1* to activate the *Re1* enhancer in a dosage-dependent manner, showing that Nkx6.1 and Isl1 regulate *Arx* antagonistically through competition at the *Re1* enhancer. However, in the presence of *Nkx6.1*, addition of *CMV-Isl1* was not sufficient to revert Nkx6.1-mediated repression (Figure 6G), indicating dominance of Nkx6.1 repressive over Isl1 activator activity. While our experiments show that Nkx6.1 is able to repress the Arx *Re1* enhancer in alpha cell lines, Nkx6.1 cannot evoke an alpha-to-beta cell fate change when misexpressed in differentiated alpha cells (Figure S3). Thus, Nkx6.1-dependent *Arx* repression through the *Re1* enhancer appears to be functionally most relevant during endocrine cell type specification, when Nkx6.1 prevents initiation of *Arx* expression (Figure 4E, 4F).

### *Nkx6.1* is necessary for maintaining beta cell identity

**Figure 7:**
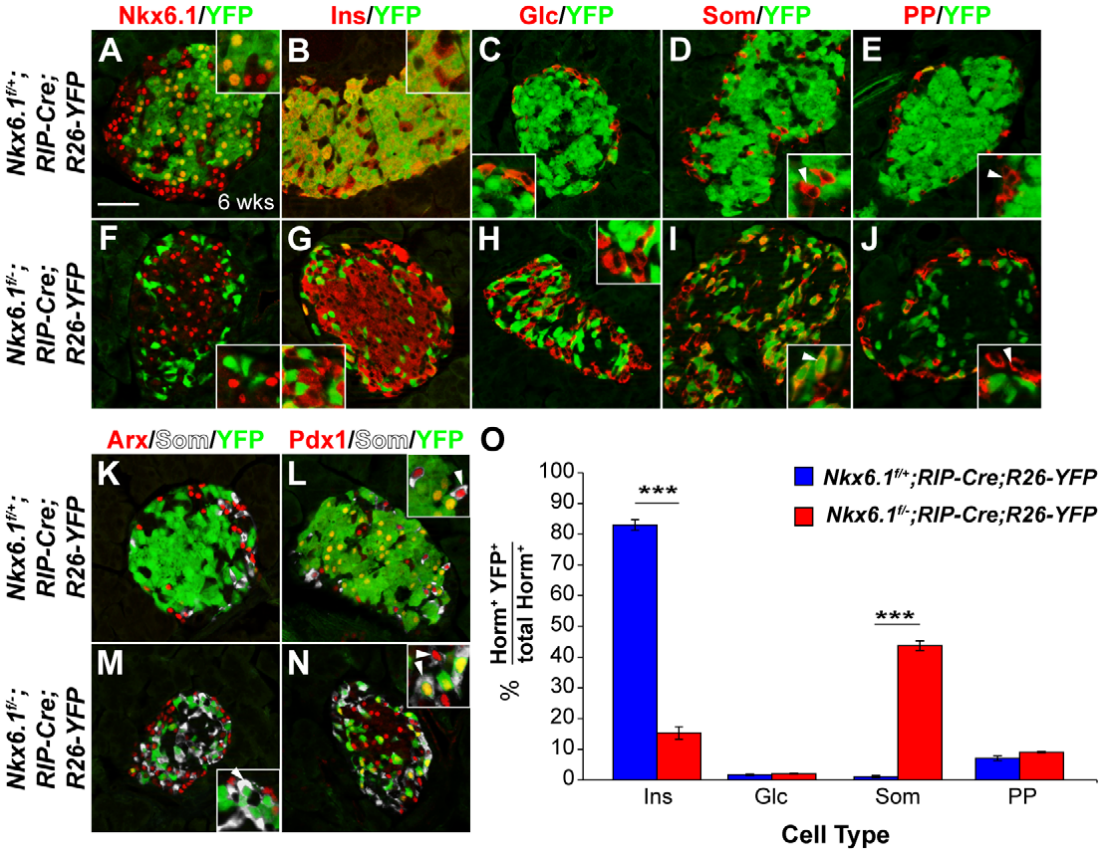
Loss of *Nkx6.1* in beta cells causes beta-to-delta cell conversion. Immunofluorescence staining of pancreata from *Nkx6.1^f/+^;RIP-Cre;R26-YFP* and *Nkx6.1^f/−^;RIP-Cre;R26-YFP* mice at 6 weeks of age shows Nkx6.1 (A) and insulin (B) expression in YFP^+^ cells of *Nkx6.1^f/+^;RIP-Cre;R26-YFP* control mice, but loss of Nkx6.1 (F) and insulin (G) in YFP^+^ cells of *Nkx6.1^f/−^;RIP-Cre;R26-YFP* mice. The insets display higher magnification images. YFP^+^ cells do not express glucagon (C, H) and rarely express pancreatic polypeptide (E, J; insets, arrowheads) in either genotype. While YFP^+^ cells are somatostatin^−^ in *Nkx6.1^f/+^;RIP-Cre;R26-YFP* mice (D; insets, arrowheads), YFP-labeled cells are mostly somatostatin^+^ in *Nkx6.1^f/−^;RIP-Cre;R26-YFP* mice (I; insets, arrowheads), suggesting beta-to-delta cell conversion. Arx expression is similar in both genotypes and absent from lineage-labeled YFP^+^ cells (K, M; inset, arrowhead), showing that loss of *Nkx6.1* in beta cells does not activate *Arx*. Pdx1^+^somatostatin^+^ cells are found in both genotypes (L, N; insets, arrowheads), but express YFP only in *Nkx6.1^f/−^;RIP-Cre;R26-YFP* mice (L; inset, arrowhead). (O) Quantification of the percentage of hormone^+^YFP^+^ cells relative to all hormone^+^ cells for each islet cell type shows reduced numbers of insulin^+^YFP^+^ cells and increased numbers of in somatostatin^+^YFP^+^ cells in *Nkx6.1^f/−^;RIP-Cre;R26-YFP* mice compared to *Nkx6.1^f/+^;RIP-Cre;R26-YFP* mice at 6 weeks (n = 3). Wks, weeks; Ins, insulin; Glc, glucagon; PP, pancreatic polypeptide; Som, somatostatin; Horm, hormones. Scale bar = 50 µm. Error bars represent S.E.M; ***p<0.0001.

It has recently been shown that Nkx2.2 is an obligatory repressor of *Arx* in differentiated beta cells and that the absence of Nkx2.2 repressor activity causes beta-to-alpha cell conversion in mice [17]. Since Nkx2.2 is expressed in both beta and alpha cells, it has been speculated that beta cell-specific repression of *Arx* might depend on Nkx6.1 [17]. To directly test this hypothesis, we deleted *Nkx6.1* selectively in beta cells, using the rat *insulin* promoter II (*RIP*)*-Cre* transgene to recombine the *Nkx6.1^f^* allele and the *Rosa26* (*R26)-YFP* reporter allele. As expected, the YFP lineage label was largely confined to beta cells in control *Nkx6.1^f/+^;RIP-Cre;R26-YFP* mice at 6 weeks of age (Figure 7A–7E, 7O). In striking contrast, only a few *Nkx6.1*-deficient, YFP-labeled cells expressed insulin in *Nkx6.1^f/−^;RIP-Cre;R26-YFP* mice (Figure 7F, 7G, 7O), suggesting that cells that once activated the *insulin* promoter no longer expressed insulin. Analysis of YFP expression in conjunction with glucagon, somatostatin, and PP revealed that *Nkx6.1*-deficient beta cells adopted delta cell identity, but did not convert into alpha or PP cells (Figure 7H–7J, 7O). Thus, loss of *Nkx6.1* in differentiated beta cells no longer causes activation of glucagon and PP, as observed after *Nkx6.1* inactivation in Ngn3^+^ endocrine precursors. These findings suggest that *Nkx6.1* is necessary to repress delta cell-specific genes in beta cells, but that expression of alpha and PP cell-specific genes are inhibited through an Nkx6.1-independent mechanism once beta cells have formed.

To further test whether *Nkx6.1* deficiency in beta cells could lead to partial activation of an alpha cell gene expression program, we examined YFP^+^ cells for Arx expression. YFP^+^ cells rarely expressed Arx in both control and *Nkx6.1^f/−^;RIP-Cre;R26-YFP* mice (Figure 7K, 7M), demonstrating that Nkx6.1 is no longer necessary for *Arx* repression after beta cells have differentiated. Likewise, Pdx1 expression in adult islet cells was Nkx6.1-independent, as somatostatin^+^ cells that arose from *Nkx6.1*-deficient insulin-expressing cells were Pdx1^+^ (Figure 7L, 7N). Our data reveal that gene regulation by Nkx6.1 is highly context-dependent. While Nkx6.1 is necessary for *Arx* repression and Pdx1 activation in beta cell precursors, both genes are regulated by Nkx6.1-independent mechanisms in mature beta cells.

## Discussion

**Figure 8:**
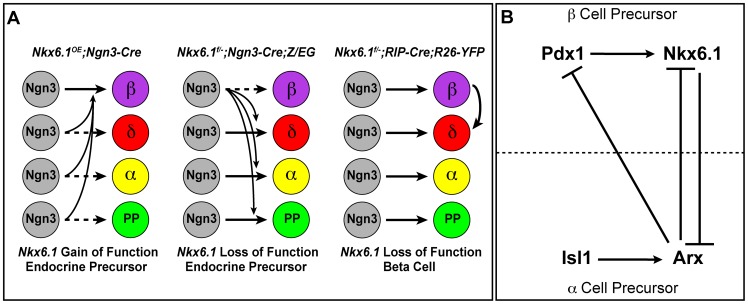
Model of Nkx6.1 function in endocrine precursor cells. (A) Expression of *Nkx6.1* results in allocation of precursors from all non-beta endocrine lineages to the beta cell lineage. Deletion of *Nkx6.1* in endocrine precursors has the opposite effect. When *Nkx6.1* is deleted in beta cells, beta cells convert into delta cells, but not into alpha or pancreatic polypeptide (PP)-producing cells. (B) Our study suggests that in endocrine precursors, Nkx6.1 and Isl1 compete for repression and activation, respectively, of the alpha cell fate determinant *Arx*. We also demonstrate that the expression of Pdx1 in endocrine precursors depends on Nkx6.1. In conjunction with previous studies, showing repression of Pdx1 and Nkx6.1 by Arx [39] and activation of Nkx6.1 by Pdx1 [34], our data support a model whereby cross-repression between Arx and Nkx6.1 confers alpha *versus* beta cell precursor identity. In beta cell precursors, Nkx6.1 expression is reinforced by Pdx1, which is repressed by Arx in alpha cell precursors.

The specification of pancreatic endocrine cell types is governed by the transcription factors Pdx1, Pax4, and Arx [12], [13], [16]. However, as none of these transcription factors control the development of solely one endocrine cell type, the molecular mechanisms that confer lineage specificity have remained largely elusive. Here, we demonstrate that the beta cell-specific transcription factor Nkx6.1 is both necessary and sufficient to specify the beta cell lineage (Figure 8A). We show that Nkx6.1 and Isl1 antagonistically regulate *Arx* expression in endocrine precursors through a direct transcriptional mechanism (Figure 8B). Thus, our study identifies Nkx6.1 as a critical beta cell programming factor that promotes the beta cell fate choice by simultaneously inducing beta cell genes and repressing non-beta endocrine genes.

### Nkx6.1: a master regulator of the beta cell fate choice

We found that forced expression of Nkx6.1 allocates endocrine precursors to the beta cell lineage. While a similar activity has been described for Pdx1 [16], Nkx6.1 and Pdx1 display different kinetics of beta cell programming. As evidenced by cells coexpressing insulin, glucagon, and Arx at birth [16], forced expression of Pdx1 in endocrine precursor cells initially produces cells with mixed alpha and beta cell identity. In contrast to *Ngn3-Cre;Pdx1^OE^* mice, we rarely observed cells exhibiting both alpha and beta cell features in neonatal *Ngn3-Cre;Nkx6.1^OE^* mice, suggesting that Nkx6.1 plays a critical role early during cell fate specification of endocrine precursors. This notion is consistent with our finding that Nkx6.1 acts as a direct transcriptional repressor of the *Arx* gene. While Pax4 has also been shown to act as a direct repressor of *Arx* [38], our observation that Pax4 expression is not affected by *Nkx6.1* ablation suggests that Pax4 alone is not sufficient to repress *Arx* in beta cell precursors. Given the dependence of Nkx6.1 expression on Pax4 activity [15], it is possible that the observed derepression of Arx and glucagon in *Pax4* mutant mice [12], [38] is a consequence of Nkx6.1-deficiency.

In addition to repressing alpha cell-specific genes through the regulation of *Arx*, Nkx6.1 also reallocated delta, PP, and epsilon cell precursors to the beta cell lineage. Likewise, inactivation of *Nkx6.1* in endocrine precursors resulted in increased production of cells of all non-beta endocrine cell types and ectopic expression of non-beta cell hormones in insulin^+^ cells. The phenotype observed upon conditional activation or deletion of *Nkx6.1* in endocrine precursors identifies Nkx6.1 as a potent general repressor of non-beta endocrine gene expression programs. While our study establishes Nkx6.1 as a direct repressor of the alpha cell fate determinant *Arx*, Nkx6.1 likely represses additional cell fate determinants critical for the specification of delta, PP, and epsilon cells. As little is known about the transcription factors mediating non-beta endocrine cell fate choices, identification of additional direct target genes for Nkx6.1 in endocrine cell fate specification will have to await future studies.

Similar to Nkx6.1, forced expression of Pax4 also conferred beta cell identity to precursors of all endocrine cell lineages [14]. However, in contrast to *Pax4* misexpressing mice [14], we did not observe oversized islets or diabetes in adult mice misexpressing *Nkx6.1*. Our observations in *Ngn3-Cre;Nkx6.1^OE^* mice are consistent with our previous study, showing that transgenic overexpression of Nkx6.1 in beta cells does not stimulate beta cell proliferation or perturb beta cell function [26]. Thus, despite their shared property as a direct repressor of *Arx* [38], Nkx6.1 and Pax4 must also have distinct targets in endocrine cells. The lack of adverse effects on beta cell function makes Nkx6.1 an excellent candidate for beta cell programming strategies.

### The transcriptional network that specifies beta cells

Unlike Pax4 expression, which we found to be independent of Nkx6.1, maintenance of Pdx1 expression downstream of *Ngn3* required Nkx6.1 activity. Previous genetic studies in mice have shown that the expression of Nkx6.1 in beta cells also depends on Pdx1 activity [34], suggesting that these two “pro-beta” transcription factors reinforce each other's expression. Since both Pdx1 and Nkx6.1 control the expression of critical genes for beta cell function, such as *MafA* and *Glut2* ([20], [25], [34]; this study), the positive regulatory loop between Pdx1 and Nkx6.1 might be critical for initiating and stabilizing beta cell-specific gene expression programs during endocrine cell differentiation. The mutual reinforcement in gene expression between these two transcription factors also explains why the combined deletion of one copy of *Pdx1* and *Nkx6.1* was sufficient to destabilize the beta cell fate choice and to cause ectopic expression of glucagon in insulin^+^ cells. Nkx6.1 could regulate *Pdx1* either directly or indirectly by repressing an inhibitor of Pdx1 expression. Since Nkx6.1 directly represses *Arx* and *Arx* has previously been shown to repress both Pdx1 and Nkx6.1 [39], loss of Pdx1 in *Nkx6.1*-deficient precursors is most likely the consequence of *Arx* derepression (Figure 8B).

### Competence window of beta cell programming

We found that expression of Nkx6.1 did not force all Ngn3^+^ cells to differentiate into beta cells and that small numbers of alpha, delta, PP, and epsilon cells expressing Nkx6.1 persisted during adulthood. This implies that the competence of precursors to adopt a beta cell fate upon *Nkx6.1* activation is limited to a short period during development when cells are still plastic and lineage-specific gene expression programs have not been fully established. Our observation that misexpression of Nkx6.1 in alpha cells failed to induce conversion of alpha into beta cells supports the notion that cells quickly lose the competence to respond to Nkx6.1 repressive cues as they undergo endocrine differentiation.

Our study shows that Nkx6.1 occupies the *Arx* enhancer in beta cells (Figure 6D), but unlike its inactivation in endocrine precursors, *Nkx6.1* inactivation in beta cells does not activate Arx. This finding suggests that Nkx6.1 is only capable of repressing *Arx* during endocrine cell differentiation. One potential mechanism that could account for Nkx6.1-independent *Arx* repression in beta cells is DNA methylation, which has recently been shown to occur at the *Arx* locus [40]. Interestingly, deletion of the DNA methyltransferase *Dnmt3a* or *Dnmt1* in beta cells results in *Arx* derepression and spontaneous conversion of beta into alpha cells [17], [40], suggesting that DNA methylation alone is sufficient to keep *Arx* repressed in beta cells. Dnmt3a is recruited to *Arx* by Nkx2.2 and loss of Nkx2.2 repressor activity causes spontaneous conversion of beta into alpha cells [17], demonstrating that Arx expression in beta cells can be readily induced when Nkx2.2 repressor function is removed. Whereas Nkx2.2 forms a complex with Dnmt3a in the 5′ regulatory region of *Arx* [17], we show that Nkx6.1 occupies an intronic *Arx* enhancer, where it competes with Isl1 and prevents Isl1 from activating *Arx*. Thus, it appears that DNA methylation-dependent repression of *Arx* is particularly important for keeping Arx repressed in beta cells, whereas Nkx6.1 prevents *Arx* activation in differentiating beta cell precursors. Overall, the role of DNA methylation in restraining cell plasticity during development is still poorly understood. Knowledge of how islet cell type-specific genes are epigenetically modified as cells differentiate and how this process can be reversed will prove important for devising effective cell programming and reprogramming strategies.

Our study highlights the importance of Nkx6.1 as a beta cell programming factor during endocrine cell differentiation and shows that insulin expression can be initiated independent of Nkx6.1 and Pdx1. Strikingly, the insulin^+^Nkx6.1^−^Pdx1^−^ cells observed after conditional inactivation of *Nkx6.1* in Ngn3^+^ cells display a similar molecular profile as insulin^+^ cells generated *in vitro* with current hESC differentiation protocols. Like *Nkx6.1*-deficient insulin^+^ cells in *Nkx6.1^f/−^;Ngn3-Cre* mice, hESC-derived insulin^+^ cells are polyhormonal and fail to express Nkx6.1, Pdx1, MafA, and critical glucose transporters ([18], [19]; Sander laboratory, unpublished data), which suggests that Nkx6.1 is required for complete beta cell programming in mice and humans. Our studies now pave the way for exploring the effectiveness of Nkx6.1 in (re)-programming strategies to generate functional beta cells for diabetes therapy.

## Materials and Methods

### *Nkx6.1* gene targeting, mice, and glucose tolerance test

*CAG-Bgeo,-Nkx6.1,-eGFP* (*Nkx6.1^OE^*) [23], *Nkx6.1^+/−^* [29], *Pdx1* ^+/−^ [41], *Neurog3* ^eGFP^ [42], *Prm1-Cre* [43], *Ngn3-Cre* [44], *RIP-Cre* [45], *Rosa26-YFP* [46], *CAG-Bgeo,-eGFP* (*Z/EG*) [47], and *Glc-Cre* mice [48] have been previously described. To create the *Nkx6.1^flox^* (*Nkx6.1^f^*) allele, a targeting vector consisting of two *loxP* sites inserted into the first and second introns of *Nkx6.1* was generated (Figure S4A). The *herpes simplex virus-thymidine kinase* gene was placed outside of the *Nkx6.1* gene homology region for negative selection. After electroporation of 129S6-derived mouse embryonic stem cells, 375 clones survived *neomycin^R^* selection. Southern blotting identified 14 clones as correctly targeted. Two clones carrying the *Nkx6.1^flox-PGK-Neo^* allele were independently injected in mouse blastocysts, and chimeric mice bred with C57BL/6J mice for germline transmission screening. The FRT-flanked *neomycin^R^* gene in intron 2 was subsequently removed by crossing *Nkx6.1^flox-PGK-Neo^* mice with *ActB-FlpE* mice (JAX; more information at http://www.mmrrc.org/strains/29994/029994.html).

Midday on the day of vaginal plug appearance was considered e0.5. Glucose tolerance tests were performed as previously described [26].

### Immunohistochemistry, morphometry, and cell quantification

Tissue preparation, immunofluorescence and TUNEL staining were performed as previously described [49]. For detection of nuclear antigens, antigen retrieval was performed in pH 6.0 citrate buffer and sections were permeabilized in 0.15% Triton X-100 in PBS. The following primary antibodies were used at the given dilutions: mouse anti-insulin (Sigma), 1∶5000; guinea pig anti-insulin (Dako), 1∶2000; guinea pig anti-glucagon (Sigma), 1∶2000; rabbit anti-PP (Dako), 1∶2000; goat anti-ghrelin (Santa Cruz), 1∶1000; rabbit anti-somatostatin (Dako), 1∶3000; goat anti-chromogranin A (Santa Cruz), 1∶500; rat anti-GFP (C. Kioussi), 1∶1000; mouse anti-Nkx6.1 (BCBC clone #2023; against C-terminal part of Nkx6.1), 1∶500; guinea pig anti-Ngn3 [24], 1∶2000; rabbit anti-Pax6 (Chemicon), 1∶1000; rabbit anti-Brn4 (M. Rosenfeld), 1∶500; rabbit anti-Ki67 (Lab Vision), 1∶500; rabbit anti-MafB (Bethyl Labs), 1∶1000; rabbit anti-MafA (Bethyl Labs), 1∶1000; rabbit anti-Pax4 (B. Sosa-Pineda), 1∶100; rabbit anti-Arx (P. Collombat), 1∶500; rabbit anti-Arx (K. Morohashi), 1∶250; guinea-pig anti-Pdx1 (C. Wright), 1∶10,000. Staining with antibodies raised in mice was conducted using the M.O.M. Kit (Vector Labs) in conjunction with streptavidin-conjugated secondary antibodies (Jackson ImmunoResearch). When necessary, nuclei were counterstained with Hoechst 33342 (Invitrogen) at 10 µg/ml. Primary antibodies were detected with donkey-raised secondary antibodies conjugated to Cy3, Cy5, DyLight488 (Jackson ImmunoResearch) or Alexa488 (Molecular Probes) at 1∶1500 dilution (1∶500 for Cy5). ApoTome images were captured on a Zeiss Axio Observer Z1 microscope with Zeiss AxioVision 4.8 and figures prepared with Adobe Photoshop/Illustrator CS4. Where necessary, the Cy5 channel was pseudo-colored white. Images were processed in accordance with the *Journal of Cell Biology* figure manipulation guidelines.

For all morphometric analyses and cell quantifications, a total of 10 sections per mouse from at least three mice per genotype were analyzed. For TUNEL, proliferation, and cell lineage analyses, the number of GFP^+^Hoechst^+^marker^+^ cells was manually counted, divided by the total number of GFP^+^Hoechst^+^ (*Ngn3-Cre*-mediated lineage tracing) or marker^+^Hoechst^+^ (*RIP-Cre*-mediated lineage tracing) cells, and multiplied by 100. For the analysis of *Pdx1* and *Nkx6.1* single and compound heterozygous mice, sections were stained for insulin, glucagon, and somatatostatin and all marker^+^ cells (on average 2100 cells per pancreas) were manually counted. Insulin^+^ cells were analyzed for the expression of glucagon or somatostatin. For quantification of GFP^+^marker^+^ cells, on average 500 GFP^+^ cells from at least five different sections per mouse were counted.

For islet cell mass measurements, images covering an entire pancreas section were tiled using a Zeiss Axio Oberver Z1 microscope with the Zeiss ApoTome module. The hormone^+^ area and total pancreas area were measured using ImagePro Plus 5.0.1 software (Media Cybernetics) and islet cell mass was calculated as follows: (hormone^+^ area/total pancreatic area) multiplied by pancreatic weight.

### Western blot, ChIP, and reporter gene assays

Western blot analysis using anti-Nkx6.1 (P. Serup) and anti-HDAC1 (Santa Cruz) antibodies was performed as previously described [26].

Based on previously identified motifs [50], [51], a custom positional weight matrix was used to identify putative Nkx6.1 binding sites. ChIP assays were performed as described [52], using Nkx6.1 (P. Serup, 1∶250) or rabbit IgG antisera. Immunoprecipitations were performed on Min6 cells (ATCC) or on 1.5×10^6^ GFP^+^ cells isolated from 300 pancreata of *Neurog3^e^* ^GFP/+^ embryos at e15.5 by fluorescence activated cell sorting. Each ChIP assay was quantified in triplicate by qPCR. The following primer sequences were used: (site A) 5′-CAT CCG GTG ATA CTG GAA GCC C -3′ and 5′-GTC TTT ATC TGA GGG GGG GCT G -3′; (site B) 5′-GCA GAG GGG GGA GGA GGG -3′ and 5′- CGG CAG GGA AAT CCA CAA AAC -3′; (site C; Re1) 5′- CCA TTT GAA GGC AAA ATG CT -3′ and 5′- GTA TGG GCT GCA AAC ACC TT -3′; (site C; Re2) 5′- TGA AGT GGC TGA ATG AGA GC -3′ and 5′- AGT TGG AGC GCG TTT TGT AG -3′; *glucagon* 5′- AAG CAG ATG AGC AAA GTG AGT G -3′ and 5′- AGG CTG TTT AGC CTT GCA GAT A -3′; and intergenic control 5′- CAC TCA GAT CCT GAG CCA CA -3′ and 5′- GCT CTC TGC CTT CCA CTT TG -3′.

The *Isl1 Re1* enhancer and *CMV-Isl1* constructs as well as procedures for transient transfections and luciferase assays have been described previously [53]. Unless indicated otherwise, 0.1 µg of *CMV-Nkx6.1* was transfected. Luciferase and Renilla expression were measured 48 hours post transfection. For each data point relative luciferase activity was quantified as the total luciferase units divided by the total Renilla units. All reporter gene analyses were performed in triplicate.

### Statistical analysis

All values are shown as mean ± standard error of the mean (SEM); *p*-values were calculated using student's 2-tailed *t*-test; *P*<0.05 was considered significant.

## Supporting Information

Figure S1 Forced *Nkx6.1* expression results in beta cell programming without altering islet cell mass. Morphometric analysis of hormone^+^ cell area at postnatal day (P) 2 (A) or islet cell mass at 5 months of age (B) shows no difference between *Ngn3-Cre;Z/EG* and *Ngn3-Cre;Nkx6.1^OE^* mice (n = 3). (C) Misexpression of *Nkx6.1* in all endocrine cell types does not alter glucose tolerance. (D–Q) Immunofluorescence staining of pancreata from *Ngn3-Cre;Z/EG* and *Ngn3-Cre;Nkx6.1^OE^* mice at P2. Quantification of the average percentage of insulin^+^GFP^+^ cells expressing the displayed marker is shown in each panel. Recombined insulin^+^GFP^+^ cells in *Ngn3-Cre;Nkx6.1^OE^* mice express the beta cell markers Pdx1 (D, E), MafA (F, G), and Pax6 (H, I) as in control *Ngn3-Cre;Z/EG* mice. Recombined, insulin^+^GFP^+^ cells rarely express the alpha cell markers Arx (J, K) and Brn4 (L, M), showing that the majority of recombined cells have no hybrid alpha/beta identity. Likewise, recombined, insulin^+^GFP^+^ cells seldom express glucagon (N, O) or somatostatin (P, Q). Arrowheads point to insulin^+^ cells that have recombined the *Nkx6.1^OE^* transgene. Ins, insulin; Glc, glucagon; Som, somatostatin. Scale bar = 50 µm. Error bars represent S.E.M.
(TIF)

Figure S2 Stable expression of Nkx6.1 in endocrine precursors and their progeny results in persistent increase of beta cells and decrease of alpha cells in adult mice. Immunofluorescence staining of pancreata from 5-month-old *Ngn3-Cre;Z/EG* and *Ngn3-Cre;Nkx6.1^OE^* mice for GFP with each of the endocrine hormones (A–H). Quantification of the percentage of lineage-labeled progeny of *Ngn3*-expressing cells that express insulin, glucagon, somatostatin, or pancreatic polypeptide at 5 months of age (I) (n = 3). Ins, insulin; Glc, glucagon; Som, somatostatin; PP, pancreatic polypeptide; Horm, hormone; mo, month. Scale bar = 50 µm. Error bars represent S.E.M.; *p<0.05.
(TIF)

Figure S3 Forced expression of *Nkx6.1* in alpha cells does not cause alpha-to-beta cell conversion. (A) Schematic of the transgenes used for conditional *Nkx6.1* misexpression and cell tracing; Triangles, *loxP* sites; Ovals, *internal ribosomal entry sites* (IRES). (B, C) Immunofluorescence staining of pancreata from *Glc-Cre;Z/EG* and *Glc-Cre;Nkx6.1^OE^* mice at 4 months of age for GFP together with glucagon (Glc) and insulin (Ins) (B) or Nkx6.1 with glucagon and insulin (C). The insets display higher magnification images. Nkx6.1 is ectopically expressed in glucagon^+^ cells, but the GFP lineage label is not detected in insulin^+^ cells. Scale bar = 50 µm.
(TIF)

Figure S4 Generation of the *Nkx6.1^flox^* allele. (A) Schematic of the gene targeting strategy to generate the *Nkx6.1^flox^* allele. Cre recombinase-mediated recombination of the two *loxP* sites removes exon 2 (closed triangles = *loxP* sites, open triangles = *Frt* sites). (B) The gross morphology of *Nkx6.1^f/−^;Prm-Cre* (*Nkx6.1^Δf/−^*) embryos at e18.5 is identical to *Nkx6.1* null mutants. (C) Western blot analysis of pancreatic lysates from e14.5 *wild type, Nkx6.1^Δf/+^*, and *Nkx6.1^Δf/−^* embryos demonstrates absence of Nkx6.1 protein in lysates from *Nkx6.1^Δf/−^* embryos. HDAC1 was used as a loading control. Immunofluorescence staining for insulin and glucagon on pancreatic sections from *wild type* (D), *Nkx6.1^Δf/+^* (E), and *Nkx6.1^Δf/−^* (F) embryos at e18.5 shows marked reduction in beta cells upon *Nkx6.1* deletion. Ins, insulin; Glc, glucagon. Scale bar = 50 µm.
(TIF)

Figure S5 Expression of Pax6 is maintained in *Nkx6.1-*deficient cells. Immunofluorescence staining for Nkx6.1 (A, B) and Pax6 (C, D) in pancreata from *Ngn3-Cre;Z/EG* and *Nkx6.1^f/−^;Ngn3-Cre;Z/EG* mice at postnatal day (P) 2 shows absence of Nkx6.1 and normal expression of Pax6 in *Nkx6.1*-deficient, recombined, insulin^+^GFP^+^ cells. Ins, insulin. Scale bar = 50 µm.
(TIF)

Figure S6 *Nkx6.1* gain- or loss-of-function does not affect Arx expression at e15.5. Immunofluorescence staining for GFP, Ngn3, and Arx on pancreata from *Ngn3-Cre;Z/EG* (A), *Ngn3-Cre;Nkx6.1^OE^* (B), and *Nkx6.1^f/−^;Ngn3-Cre;Z/EG* (C) mouse embryos at e15.5 reveals a small subset of GFP^+^ cells expressing Ngn3 (arrowheads in A″ and B″), but no coexpression of Arx and Ngn3 (A″″, B″″, C″″). In *Ngn3-Cre;Nkx6.1^OE^* and *Nkx6.1^f/−^;Ngn3-Cre;Z/EG* mice GFP^+^ cells express Arx (B′″″, C′″″). (D) Quantification of the percentage of lineage-labeled *Ngn3*-expressing cells that express Arx in *Ngn3-Cre;Z/EG*, *Ngn3-Cre;Nkx6.1^OE^*, and *Nkx6.1^f/−^;Ngn3-Cre;Z/EG* mice at e15.5 (n = 3). Scale bar = 50 µm.
(TIF)
