# Supplementary figures and images for: Nkx6.1 Controls a Gene Regulatory Network Required for Establishing and Maintaining Pancreatic Beta Cell Identity
Source: PLoS Genet. 2013 Jan 31;9(1):e1003274. doi: 10.1371/journal.pgen.1003274 (PMC3561089; doi:10.1371/journal.pgen.1003274)

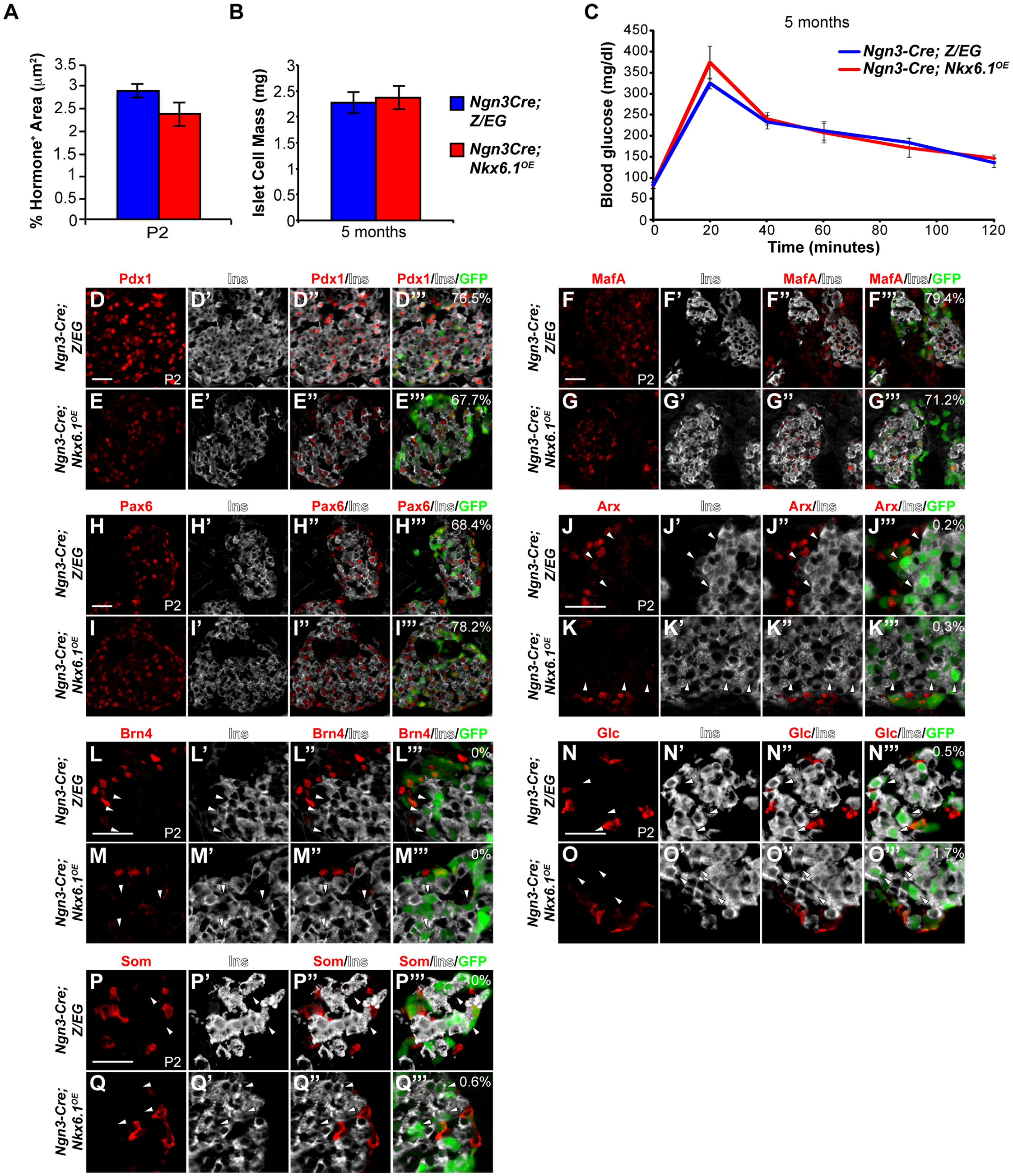

Supplement: Figure S1 — Forced Nkx6.1 expression results in beta cell programming without altering islet cell mass. Morphometric analysis of hormone+ cell area at postnatal day (P) 2 (A) or islet cell mass at 5 months of age (B) shows no difference between Ngn3-Cre;Z/EG and Ngn3-Cre;Nkx6.1OE mice (n = 3). (C) Misexpression of Nkx6.1 in all endocrine cell types does not alter glucose tolerance. (D–Q) Immunofluorescence staining of pancreata from Ngn3-Cre;Z/EG and Ngn3-Cre;Nkx6.1OE mice at P2. Quantification of the average percentage of insulin+GFP+ cells expressing the displayed marker is shown in each panel. Recombined insulin+GFP+ cells in Ngn3-Cre;Nkx6.1OE mice express the beta cell markers Pdx1 (D, E), MafA (F, G), and Pax6 (H, I) as in control Ngn3-Cre;Z/EG mice. Recombined, insulin+GFP+ cells rarely express the alpha cell markers Arx (J, K) and Brn4 (L, M), showing that the majority of recombined cells have no hybrid alpha/beta identity. Likewise, recombined, insulin+GFP+ cells seldom express glucagon (N, O) or somatostatin (P, Q). Arrowheads point to insulin+ cells that have recombined the Nkx6.1OE transgene. Ins, insulin; Glc, glucagon; Som, somatostatin. Scale bar = 50 µm. Error bars represent S.E.M. (TIF) [file pgen.1003274.s001.tif]

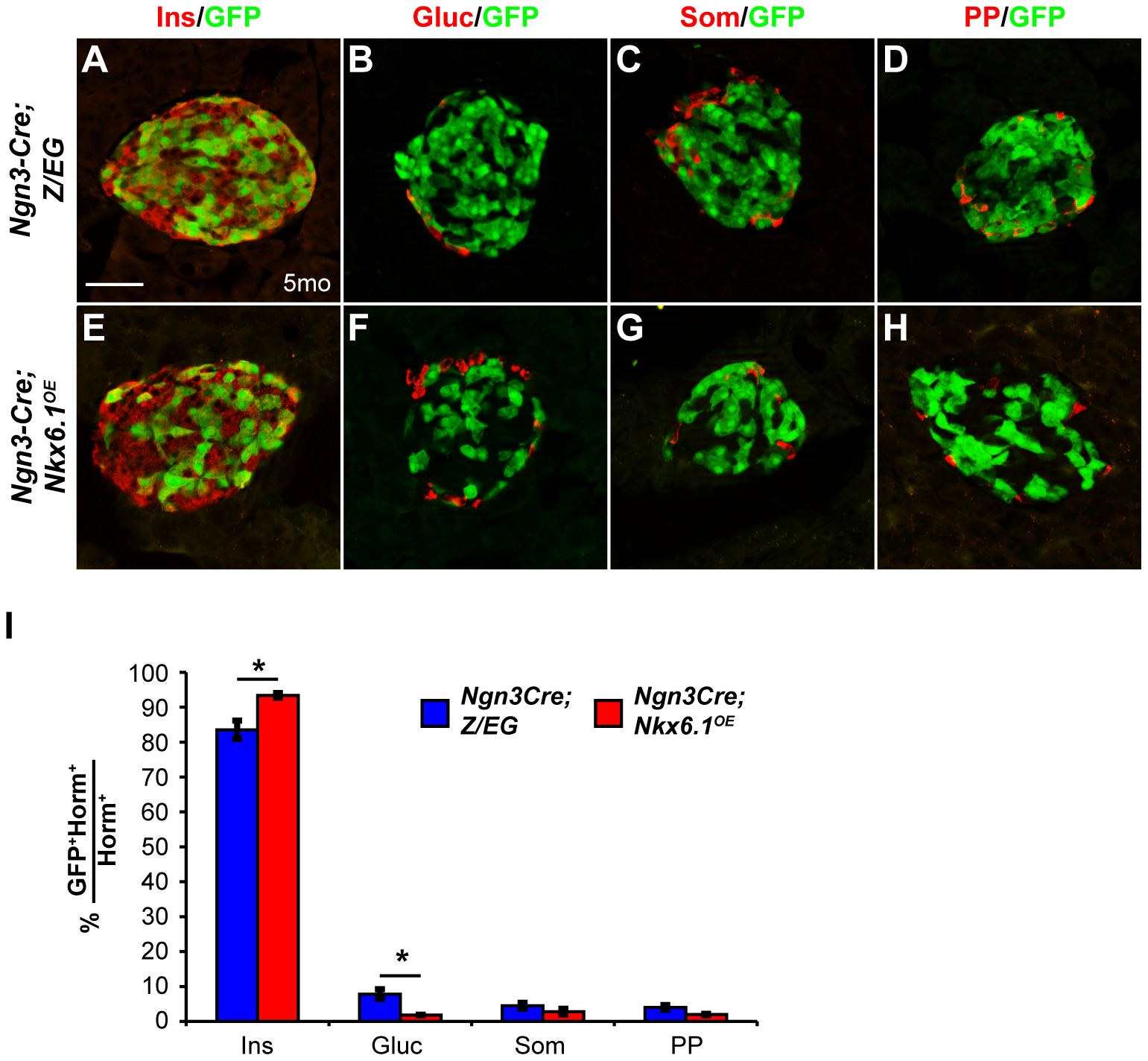

Supplement: Figure S2 — Stable expression of Nkx6.1 in endocrine precursors and their progeny results in persistent increase of beta cells and decrease of alpha cells in adult mice. Immunofluorescence staining of pancreata from 5-month-old Ngn3-Cre;Z/EG and Ngn3-Cre;Nkx6.1OE mice for GFP with each of the endocrine hormones (A–H). Quantification of the percentage of lineage-labeled progeny of Ngn3-expressing cells that express insulin, glucagon, somatostatin, or pancreatic polypeptide at 5 months of age (I) (n = 3). Ins, insulin; Glc, glucagon; Som, somatostatin; PP, pancreatic polypeptide; Horm, hormone; mo, month. Scale bar = 50 µm. Error bars represent S.E.M.; *p<0.05. (TIF) [file pgen.1003274.s002.tif]

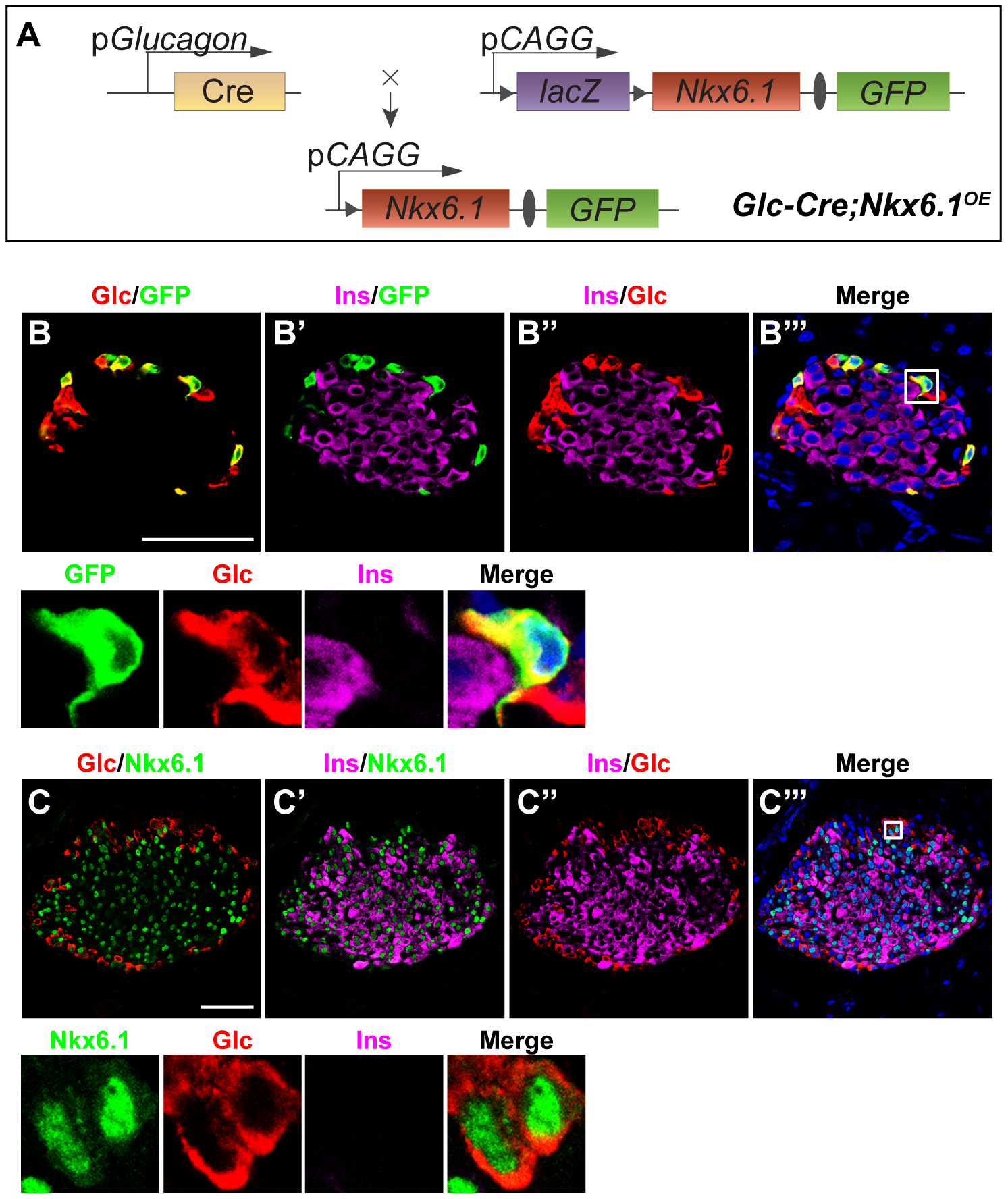

Supplement: Figure S3 — Forced expression of Nkx6.1 in alpha cells does not cause alpha-to-beta cell conversion. (A) Schematic of the transgenes used for conditional Nkx6.1 misexpression and cell tracing; Triangles, loxP sites; Ovals, internal ribosomal entry sites (IRES). (B, C) Immunofluorescence staining of pancreata from Glc-Cre;Z/EG and Glc-Cre;Nkx6.1OE mice at 4 months of age for GFP together with glucagon (Glc) and insulin (Ins) (B) or Nkx6.1 with glucagon and insulin (C). The insets display higher magnification images. Nkx6.1 is ectopically expressed in glucagon+ cells, but the GFP lineage label is not detected in insulin+ cells. Scale bar = 50 µm. (TIF) [file pgen.1003274.s003.tif]

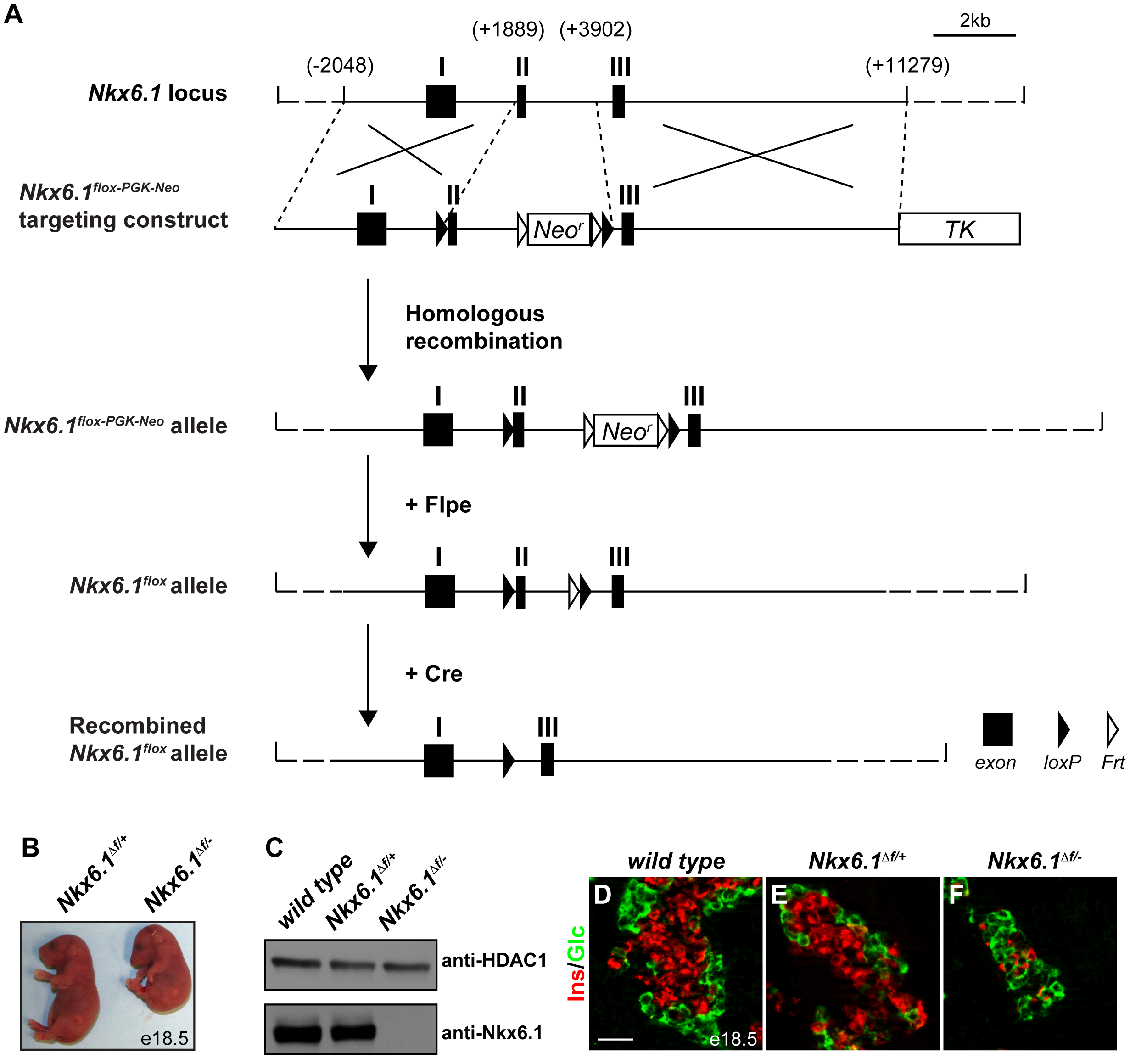

Supplement: Figure S4 — Generation of the Nkx6.1flox allele. (A) Schematic of the gene targeting strategy to generate the Nkx6.1flox allele. Cre recombinase-mediated recombination of the two loxP sites removes exon 2 (closed triangles = loxP sites, open triangles = Frt sites). (B) The gross morphology of Nkx6.1f/−;Prm-Cre (Nkx6.1Δf/−) embryos at e18.5 is identical to Nkx6.1 null mutants. (C) Western blot analysis of pancreatic lysates from e14.5 wild type, Nkx6.1Δf/+, and Nkx6.1Δf/− embryos demonstrates absence of Nkx6.1 protein in lysates from Nkx6.1Δf/− embryos. HDAC1 was used as a loading control. Immunofluorescence staining for insulin and glucagon on pancreatic sections from wild type (D), Nkx6.1Δf/+ (E), and Nkx6.1Δf/− (F) embryos at e18.5 shows marked reduction in beta cells upon Nkx6.1 deletion. Ins, insulin; Glc, glucagon. Scale bar = 50 µm. (TIF) [file pgen.1003274.s004.tif]

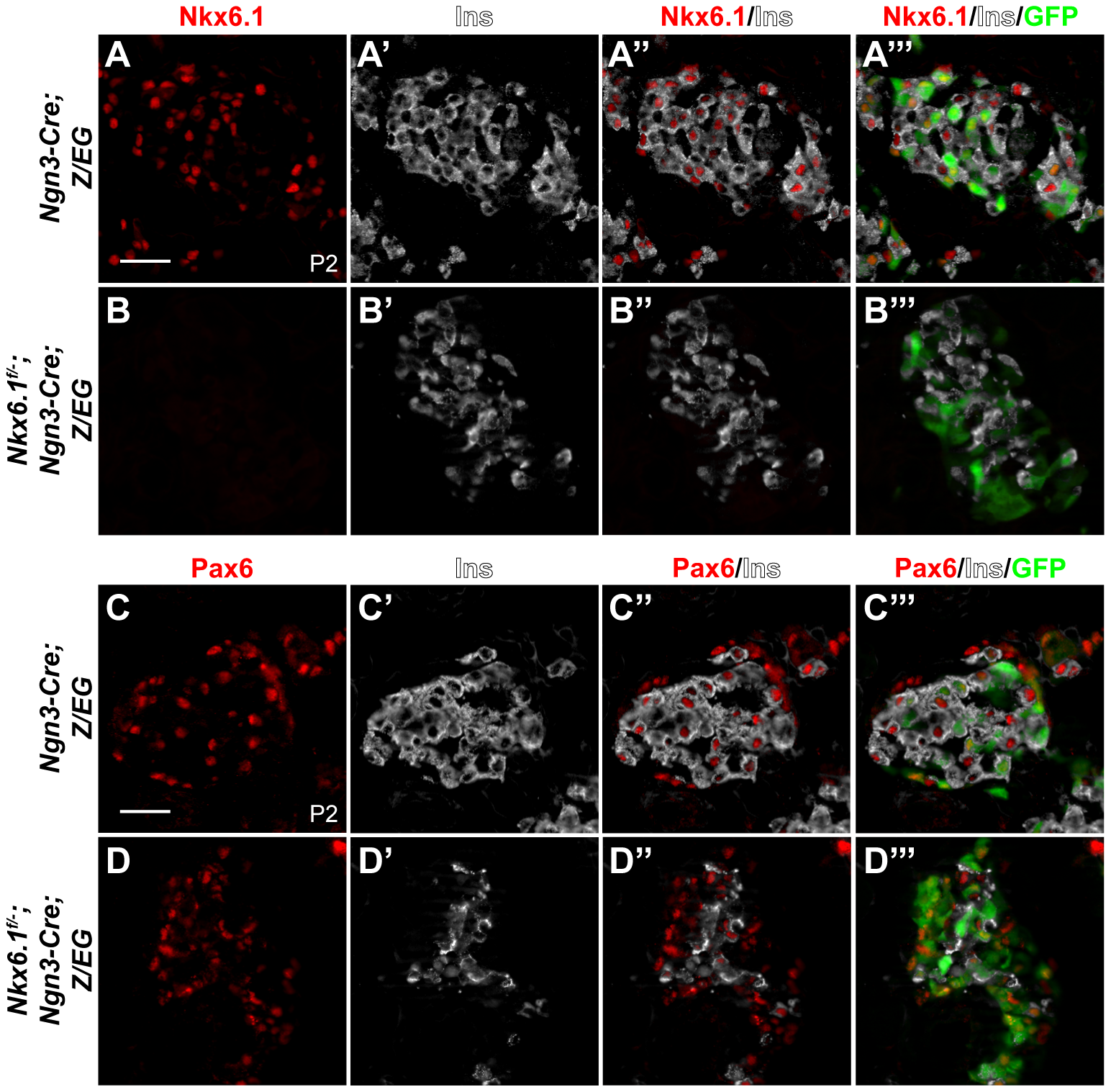

Supplement: Figure S5 — Expression of Pax6 is maintained in Nkx6.1-deficient cells. Immunofluorescence staining for Nkx6.1 (A, B) and Pax6 (C, D) in pancreata from Ngn3-Cre;Z/EG and Nkx6.1f/−;Ngn3-Cre;Z/EG mice at postnatal day (P) 2 shows absence of Nkx6.1 and normal expression of Pax6 in Nkx6.1-deficient, recombined, insulin+GFP+ cells. Ins, insulin. Scale bar = 50 µm. (TIF) [file pgen.1003274.s005.tif]

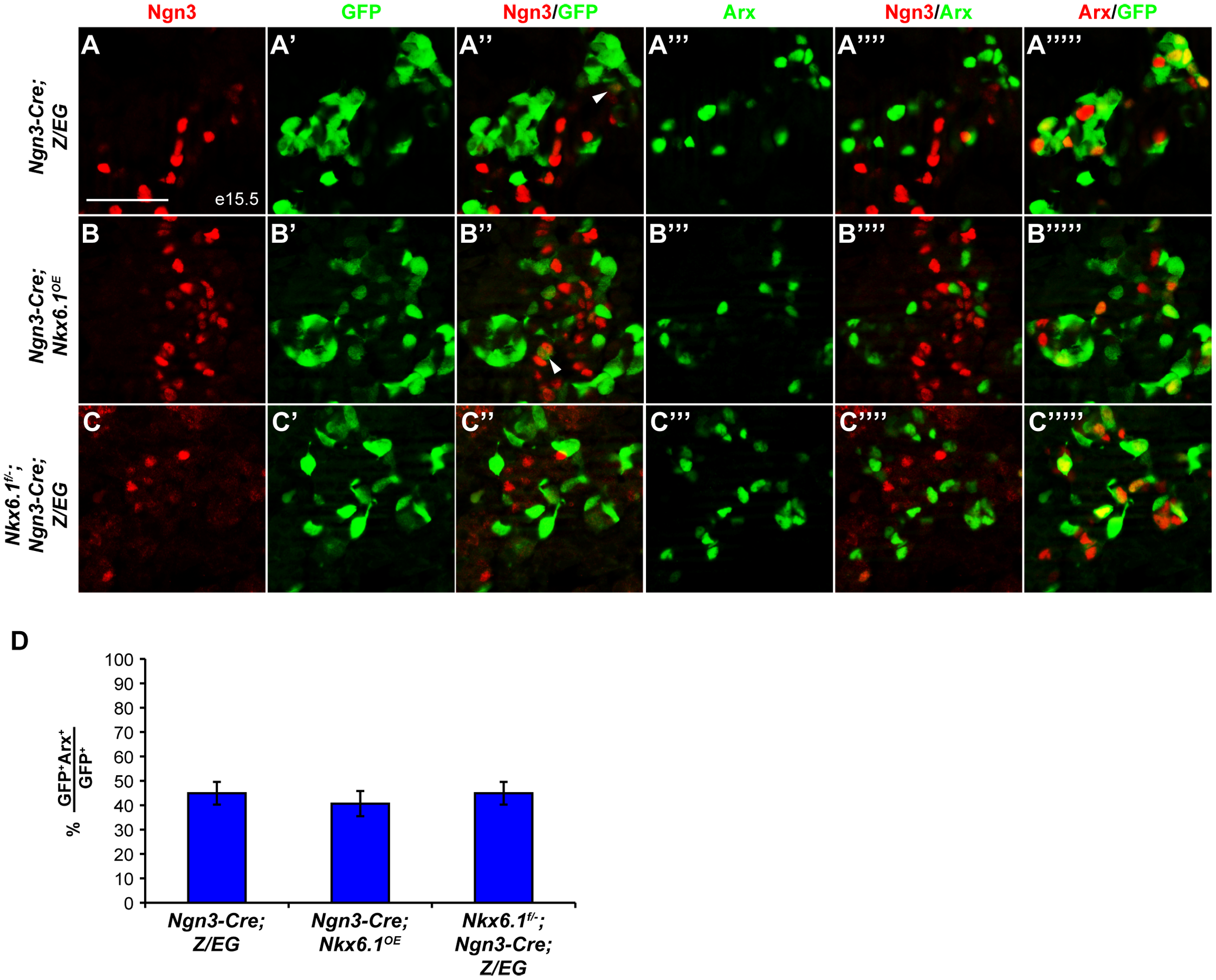

Supplement: Figure S6 — Nkx6.1 gain- or loss-of-function does not affect Arx expression at e15.5. Immunofluorescence staining for GFP, Ngn3, and Arx on pancreata from Ngn3-Cre;Z/EG (A), Ngn3-Cre;Nkx6.1OE (B), and Nkx6.1f/−;Ngn3-Cre;Z/EG (C) mouse embryos at e15.5 reveals a small subset of GFP+ cells expressing Ngn3 (arrowheads in A″ and B″), but no coexpression of Arx and Ngn3 (A″″, B″″, C″″). In Ngn3-Cre;Nkx6.1OE and Nkx6.1f/−;Ngn3-Cre;Z/EG mice GFP+ cells express Arx (B′″″, C′″″). (D) Quantification of the percentage of lineage-labeled Ngn3-expressing cells that express Arx in Ngn3-Cre;Z/EG, Ngn3-Cre;Nkx6.1OE, and Nkx6.1f/−;Ngn3-Cre;Z/EG mice at e15.5 (n = 3). Scale bar = 50 µm. (TIF) [file pgen.1003274.s006.tif]
